# Self-Reported SARS-CoV-2 Infections among National Blood Donor Cohort, United States, 2020–2022

**DOI:** 10.3201/eid3105.241953

**Published:** 2025-05

**Authors:** Bryan R. Spencer, Akintunde Akinseye, Eduard Grebe, Mars Stone, Karla G. Zurita, David J. Wright, James M. Haynes, Susan L. Stramer, Michael P. Busch

**Affiliations:** American Red Cross, Dedham, Massachusetts, USA (B.R. Spencer, J.M. Haynes); Westat, Rockville, Maryland, USA (A. Akinseye, D.J. Wright); Vitalant Research Institute, San Francisco, California, USA (E. Grebe, M. Stone, K.G. Zurita, M.P. Busch); Infectious Disease Consultant, North Potomac, Maryland, USA (S.L. Stramer)

**Keywords:** viruses, SARS-CoV-2, COVID-19, respiratory infections, public health surveillance, blood donors, health surveys, United States

## Abstract

SARS-CoV-2 case surveillance in the United States did not distinguish first infections from reinfections. In a large blood donor cohort, self-reported first infections and reinfections during 2020–2022 mirrored public health case count surveillance, and reinfection incidence peaked in 2022. Blood donor data could aid in SARS-CoV-2 and emerging infectious disease surveillance.

During the COVID-19 pandemic in the United States, case counts, test positivity rates, hospitalizations, number of deaths, wastewater surveillance, genomic surveillance, and serologic testing from blood donor populations and commercial laboratories provided surveillance information for SARS-CoV-2, but none of those methods distinguished reinfection from first infection (*1*–*6*). A further challenge to public health surveillance was widespread adoption of rapid antigen tests conducted at home; infections detected outside laboratory or healthcare settings remained outside the national surveillance system (*7*). We leveraged self-reports from a large blood donor cohort to estimate first infection and reinfection incidence during 2020–2022.

## The Study

Two large US blood collectors, American Red Cross (https://www.redcross.org) and Vitalant (https://www.vitalant.org), established a prospective cohort of 142,599 repeat blood donors to support serosurveillance of SARS-CoV-2 transmission during 2022 and to assess serologic correlates of protection against infection and illness. We used serologic status and reported COVID-19 vaccine history as of July 2021 to classify blood donors after blood center testing for spike and nucleocapsid (N) antibodies of all blood donations during June 2020–June 2021. Together, American Red Cross and Vitalant collect blood in >40 states and account for ≈45% of the US blood supply. The Advarra (https://www.advarra.com, approval no. Pro00056783) and American Red Cross (approval no. 2021-033) institutional review boards approved the study activities.

Both blood centers electronically distributed an index survey to cohort members whose donor record confirmed acceptance of email contact. Vitalant also reached out to cohort members accepting postal mail contact. Harmonized survey content captured self-reported SARS-CoV-2 infections at time of donor response that had been swab confirmed or diagnosed by a healthcare provider, date of diagnosis, attendant symptoms, and hospitalization (Appendix). We distributed index surveys and automated reminders to encourage survey completion during December 2021–December 2022. We distributed follow-up surveys capturing new infections at quarterly intervals through January 2023.

The principal outcomes of interest were survey-reported first or second infections (i.e., first reinfection) that were swab-confirmed or diagnosed by a healthcare provider. Second infections were counted only if reported >90 days after the first infection. First and second infection rates were tabulated on a quarterly basis and estimated as the infection count divided by the population at risk. We defined the population at risk for first infection for a given quarter as the participants who completed a survey during or after that quarter and who had not reported any infection before that quarter. We similarly calculated the population at risk for second infection, and participants counted toward the population at risk in the quarters after the first infection if they completed a survey during or after those quarters. We only tabulated the first reinfection for that effort; donors were censored for any quarter after their second infection or their last survey completion. Hence, donors completing at least an index survey might have been informative for population at risk for 8–12 quarters starting in January 2020.

We did not conduct modeling; we only described counts and rates estimated and plotted to show temporal trends in reported infections from the first quarter of 2020 through the fourth quarter of 2022. We combined data across the 2 blood centers and reported without weighting after observing comparable trends in both centers. We compared survey respondents with the underlying cohort in qualitative terms for assessment of the representativeness of data reported here. Comparisons to public health case surveillance were also qualitative in nature; we did not conduct statistical tests.

Our cohort included 66,274 donors (46.5% of the cohort) who completed an index survey (Table 1); of those, 46,292 (70%) completed a survey during or after the fourth quarter of 2022. Among survey respondents, 55% were women and 45% were men, and most (74%) were >50 years of age, self-reported race as non-Hispanic White (>90%), and were donors to the American Red Cross (79%). Based on self-reported vaccine status and serologic testing in mid-2021, 72% of those who completed an index survey had received a COVID-19 vaccine, 25% had N antibodies indicating prior infection, 16% were both previously infected and vaccinated, and 18% were neither vaccinated nor previously infected at cohort formation. Among survey nonrespondents, the corresponding percentages were as follows: 50% had received a vaccine, 21% had N antibodies, 12.5% had hybrid immunity, and 41.5% were unexposed at cohort formation. Compared with the full cohort, younger donors (age groups 16–29 and 30–49 years) were underrepresented and older donors (age >65 years) were overrepresented among survey respondents. Survey respondents showed modest variation relative to the underlying cohort in terms of sex, race, and ethnicity distribution, but participation varied by infection and vaccine history. Among donors with previous SARS-CoV-2 infection at time of cohort formation, vaccine history was similar in survey respondents compared with the full cohort. In contrast, among those who had not been infected by mid-2021, unvaccinated donors were less likely and vaccinated donors were more likely to complete surveys.

**Table 1:** Demographic description of underlying national blood donor cohort and survey responders for self-reported SARS-CoV-2 infections among national blood donor cohort, United States, 2020–2022

Demographic data	Full cohort, no. (%)	Completed index survey, no. (%)
All	142,599 (100%)	66,274 (46.5%)
Sex		
F	74,561 (52.3)	36,659 (55.3)
M	68,038 (47.7)	29,615 (44.7)
Age group, y		
16–29	12,357 (8.7)	2,468 (3.7)
30–49	39,054 (27.4)	14,733 (22.2)
50–64	52,507 (36.8)	26,061 (39.3)
>65	38,681 (27.1)	23,012 (34.7)
Race/ethnicity		
Native American/Alaskan, non-Hispanic	795 (0.6)	210 (0.3)
Asian/Pacific Islander, non-Hispanic	3,816 (2.7)	1,308 (2.0)
Black, non-Hispanic	2,339 (1.6)	797 (1.2)
Hispanic	12,983 (9.1)	3,004 (4.5)
Other/mixed, non-Hispanic	1,518 (1.1)	511 (0.8)
Missing/unknown/refused	2,458 (1.7)	554 (0.8)
White, non-Hispanic	118,690 (83.2)	59,890 (90.4)
Cohort assignment, July 2021		
Infected, not vaccinated	12,751 (8.9)	6,318 (9.5)
Infected, vaccinated	19,969 (14.0)	10,440 (15.8)
Not infected, not vaccinated	43,887 (30.8)	12,196 (18.4)
Not infected, vaccinated	65,992 (46.3)	37,320 (56.3)
Blood collection organization		
American Red Cross	77,075 (54.1)	52,439 (79.1)
Vitalant	65,524 (45.9)	13,835 (20.9)

Donors reported 25,973 first and 3,088 second infections during 2020–2022 (Table 2). Peak rates of first infections were observed in the fourth quarter of 2020 (4,684 cases; 72.9 cases/1,000 population at risk) and in the third quarter of 2022 (3,672 cases; 94.2 cases/1,000 at risk) and had a nadir of 170 cases (2.9 cases/1,000 population at risk) during the second quarter of 2021 (Table 2; Figure, panel A). Second infections represented 10.6% of the 29,061 combined first and second infections in aggregate, and the percentages of reported infections that were second infections varied from 0% to 1% during 2020 and from 13% to 20% during 2022 (Figure, panel B). Adjusting for population at risk, rates of second infection remained low (<2% per quarter) during the pre-Delta period (through June 2021) but surged in the second half of 2021 and almost reached parity with first infection rates during the Omicron wave in quarter 1 of 2022 (72.2 vs. 69.8 cases/1,000 population at risk) (Figure, panel A). Temporal increases and decreases in second infection rates largely mirrored the trends in first infection rates until 2022, diverged during the second and third quarters, and decreased in parallel during the fourth quarter. Combined first and second infection rates (Figure, panel C) yielded a curve that largely mirrored Centers for Disease Control and Prevention case counts grouped into quarterly bins (Figure, panel D): peak in late 2020, nadir in mid-2021, and surge in second half of 2021 during emergence of Delta that continued with the Omicron period. We saw the most notable difference in 2022, where our donor population reported a continuing increase in infections that peaked only in the third quarter, whereas public health surveillance showed a sharp decrease between the first and second quarters of 2022.

**Table 2:** Self-reported SARS-CoV-2 first and second infections by quarter among national blood donor cohort, United States, 2020–2022*

Infection measure	2020					2021					2022			
	Q1	Q2	Q3	Q4		Q1	Q2	Q3	Q4		Q1	Q2	Q3	Q4
First infection count	549	432	1,043	4,684		1,242	170	1,494	3,102		3,822	3,807	3,672	1,956
At-risk population	66,274	65,725	65,293	64,250		59,566	58,324	58,154	56,660		52,913	43,587	38,995	30,133
First infection per 1,000 population at risk, rate	8.3	6.6	16.0	72.9		20.9	2.9	25.7	54.8		72.2	87.3	94.2	64.9
Second infection count	0	1	6	33		50	29	68	322		841	545	717	476
At-risk population	0	549	980	2,017		6,668	7,860	8,001	9,427		12,050	13,022	15,935	16,159
Second infection per 1,000 population at risk, rate	0	1.8	6.1	16.4		7.5	3.7	8.5	34.2		69.8	41.9	45.0	29.5

*Q, quarter.

**Figure:**
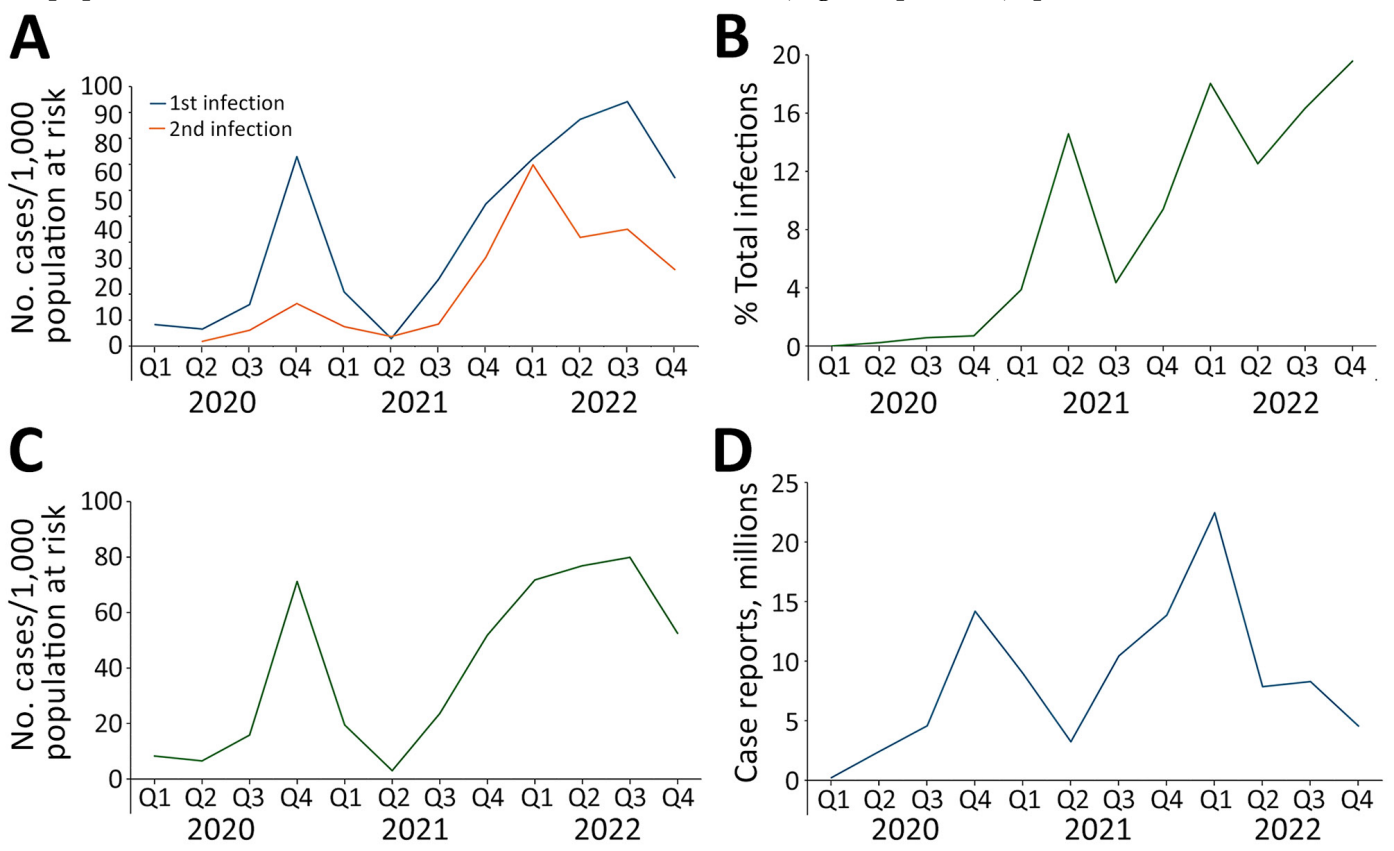
Survey of self-reported SARS-CoV-2 infections among national blood donor cohort, United States, 2020–2022. A–C) Swab-confirmed or healthcare provider–diagnosed infections grouped into quarterly bins: US blood donor survey-reported first and second COVID-19 infection incidence (A); reinfections as percentage of total infections (B); and total infection incidence (C). D) Quarterly counts of case reports through the US surveillance system.

## Conclusions

Despite the limitation that our analysis excludes infections not confirmed by a swab test or healthcare provider diagnosis (i.e., asymptomatic or mild infections for which testing was not performed), the temporal trends and rates of SARS-CoV-2 infection reported by a cohort of US blood donors are broadly consistent with public health surveillance case counts during the first 3 years of the COVID-19 pandemic, reinforcing a 2024 survey-based report (*8*). The ability to distinguish first infections from reinfections provides utility in the context of ongoing transmission, when rates of severe illness, hospitalization, and death are lower than initial waves. Although known to be healthier on average than the general population, blood donors nonetheless represent an informative population for monitoring SARS-CoV-2 and potentially other emerging infectious diseases. Blood donor data could aid in SARS-CoV-2 and emerging infectious disease surveillance.

Appendix Surveys used for self-reported SARS-CoV-2 infections among national blood donor cohort, United States, 2020–2022.
