# Supplementary material for: Self-Reported SARS-CoV-2 Infections among National Blood Donor Cohort, United States, 2020–2022
Source: Emerg Infect Dis. 2025 May;31(5):1006–9. doi: 10.3201/eid3105.241953 (PMC12044225; doi:10.3201/eid3105.241953)
Supplement: Appendix — Surveys used for self-reported SARS-CoV-2 infections among national blood donor cohort, United States, 2020–2022. [file 24-1953-Techapp-s1.pdf]

Article DOI: https://doi.org/10.3201/eid3105.241953

*EID cannot ensure accessibility for supplementary materials supplied by authors. Readers who have difficulty accessing supplementary content should contact the authors for assistance.*

# Self-Reported SARS-CoV-2 Infections among National Blood Donor Cohort, United States, 2020–2022

## Appendix

The following pages show the baseline and follow-up surveys from the American Red Cross and the baseline and follow-up surveys from Vitalant used to collect self-reported SARS-CoV-2 infections among national blood donor cohort.

## American Red Cross COVID-19 Follow-Up Study v4.01

**Thank you for clicking through to learn more about our American Red Cross (ARC) survey!**

**You are eligible for this survey because you donated between June 15, 2020 and December 31, 2021.**

**We are inviting you to participate in a follow-up survey on COVID-19. The purpose of the current survey is to better understand the impact of the pandemic and COVID-19 infection on donor health. If you have previously completed one of our surveys on COVID-19, your participation in this survey is still valuable to help answer new scientific questions.**

**This research is being conducted in collaboration with the Centers for Disease Control and Prevention. Your participation is voluntary, and there will be no impact to your relationship with American Red Cross if you decide not to participate. Please note that information that can be linked to you will not be shared outside of ARC. If you agree to participate, please begin the survey below.**

**If you have questions or concerns, please contact us Monday through Friday, 10am to 6pm Eastern time at 866-628-9875.**

1. Since March 2020, have you been infected by SARS-CoV-2, the virus that causes COVID-19?
(*If you have been infected more than once, please answer for your earliest infection*).

- ◯ Yes, and my infection was confirmed by a diagnostic test (nose swab, throat swab, saliva test) or a healthcare provider (or both)
- ◯ Yes, but my infection was confirmed only by a blood test (antibody test, such as from Red Cross)
- ◯ Yes, I think I have been infected but it has not been confirmed by a test or diagnosis
- ◯ No
- ◯ I don't know/am unsure

## American Red Cross COVID-19 Follow-Up Study v4.01

2. Do you think you have been infected by the COVID-19 virus more than once?

- ◯ Yes, twice
- ◯ Yes, three times
- ◯ No
- ◯ I don't know/am unsure

3. When were you first infected by the COVID-19 virus?
Please approximate if unsure.

| | Month | Day | Year |
|---|---|---|---|
| Date of first COVID-19 infection | | | |

4. When you were first infected by the COVID-19 virus, did you have any symptoms?

- ◯ Yes
- ◯ No
- ◯ I don't know/am unsure

American Red Cross

## American Red Cross COVID-19 Follow-Up Study v4.01

5. In the 2 weeks after your COVID-19 symptoms started, which of the following symptoms did you have? (This refers to the first time you were infected with the COVID-19 virus). Mark all that apply.

- ☐ Fever
- ☐ Chills or rigors (shaking chills)
- ☐ Muscle aches
- ☐ Headache
- ☐ Sore throat
- ☐ Nausea or vomiting
- ☐ Diarrhea
- ☐ Fatigue
- ☐ Congestion or runny nose
- ☐ Cough
- ☐ Shortness of breath or difficulty breathing
- ☐ Loss of smell
- ☐ Loss of taste
- ☐ Confusion
- ☐ Persistent chest pain or chest pressure
- ☐ Pale, gray, or blue-colored skin, lips or nail beds
- ☐ Difficulty waking up or staying awake
- ☐ Other (please specify)

6. Were you hospitalized for COVID-19 for your first infection? Choose the answer that best applies.

- Yes, I was hospitalized and admitted to the intensive care unit
- Yes, I was hospitalized but not admitted to the intensive care unit
- No, I was not hospitalized
- I don't know/am unsure

American Red Cross

## American Red Cross COVID-19 Follow-Up Study v4.01

7. When do you think you were infected by the COVID-19 virus for the second time? Please approximate if unsure.

| | Month | Day | Year |
|---|---|---|---|
| Date of second COVID-19 infection | | | |

8. Was your second infection from the COVID-19 virus confirmed?

- Yes, and my infection was confirmed by a diagnostic test (nose swab, throat swab, saliva test) or a healthcare provider (or both)
- No. I think I have been infected but it has not been confirmed by a test or diagnosis.
- I don't know/am unsure

9. When you had your second infection with the COVID-19 virus, did you have any symptoms?

- Yes
- No
- I don't know/am unsure

American Red Cross

American Red Cross COVID-19 Follow-Up Study v4.01

10. In the 2 weeks after your COVID-19 symptoms started, which of the following symptoms did you have? (This refers to the second time you were infected with the COVID-19 virus). Mark all that apply.

- ☐ Fever
- ☐ Chills or rigors (shaking chills)
- ☐ Muscle aches
- ☐ Headache
- ☐ Sore throat
- ☐ Nausea or vomiting
- ☐ Diarrhea
- ☐ Fatigue
- ☐ Congestion or runny nose
- ☐ Cough
- ☐ Shortness of breath or difficulty breathing
- ☐ Loss of smell
- ☐ Loss of taste
- ☐ Confusion
- ☐ Persistent chest pain or chest pressure
- ☐ Pale, gray, or blue-colored skin, lips or nail beds
- ☐ Difficulty waking up or staying awake
- ☐ Other (please specify)

11. Were you hospitalized for your second COVID-19 infection? Choose the answer that best applies.

- ○ Yes, I was hospitalized and admitted to the intensive care unit
- ○ Yes, I was hospitalized but not admitted to the intensive care unit
- ○ No, I was not hospitalized
- ○ I don't know/am unsure

American Red Cross

## American Red Cross COVID-19 Follow-Up Study v4.01

12. When do you think you were infected by the COVID-19 virus for the third time?
Please approximate if unsure.

| | Month | Day | Year |
|---|---|---|---|
| Date of third COVID-19 infection | | | |

13. Was your third infection from the COVID-19 virus confirmed?

- ○ Yes, and my infection was confirmed by a diagnostic test (nose swab, throat swab, saliva test) or a healthcare provider (or both)
- ○ No. I think I have been infected but it has not been confirmed by a test or diagnosis.
- ○ I don't know/am unsure

14. When you had your third infection with the COVID-19 virus, did you have any symptoms?

- ◯ Yes
- ◯ No
- ◯ I don't know/am unsure

American Red Cross

## American Red Cross COVID-19 Follow-Up Study v4.01

15. In the 2 weeks after your COVID-19 symptoms started, which of the following symptoms did you have? (This refers to the third time you were infected with the COVID-19 virus). Mark all that apply.

- ☐ Fever
- ☐ Chills or rigors (shaking chills)
- ☐ Muscle aches
- ☐ Headache
- ☐ Sore throat
- ☐ Nausea or vomiting
- ☐ Diarrhea
- ☐ Fatigue
- ☐ Congestion or runny nose
- ☐ Cough
- ☐ Shortness of breath or difficulty breathing
- ☐ Loss of smell
- ☐ Loss of taste
- ☐ Confusion
- ☐ Persistent chest pain or chest pressure
- ☐ Pale, gray, or blue-colored skin, lips or nail beds
- ☐ Difficulty waking up or staying awake
- ☐ Other (please specify)

16. Were you hospitalized for your third COVID-19 infection? Choose the answer that best applies.

- ◯ Yes, I was hospitalized and admitted to the intensive care unit
- ◯ Yes, I was hospitalized but not admitted to the intensive care unit
- ◯ No, I was not hospitalized
- ◯ I don't know/am unsure

American Red Cross

17. Prior to the COVID-19 pandemic (March 2020), did a health care professional ever tell you that you had any of these chronic health conditions? Mark all that apply.

- ☐ Chronic pain
- ☐ Anxiety
- ☐ Depression
- ☐ Chronic headaches or migraines
- ☐ Stroke
- ☐ Kidney disease
- ☐ Liver disease
- ☐ Heart disease or other cardiovascular disease
- ☐ Lung disease/asthma
- ☐ High blood pressure
- ☐ Diabetes
- ☐ Any immune system disorder
- ☐ Cancer
- ☐ NONE OF THESE

American Red Cross

American Red Cross COVID-19 Follow-Up Study v4.01

18. Have you received any COVID-19 vaccines, including boosters?

- ○ Yes, one dose
- ○ Yes, two doses
- ○ Yes, three doses
- ○ Yes, four doses
- ○ No, but I plan to
- ○ No, and I don't plan to
- ○ I don't know/am unsure

American Red Cross

American Red Cross COVID-19 Follow-Up Study v4.01

19. For your first vaccine dose, which COVID-19 vaccine did you receive?

- Pfizer-BioNTech
- AstraZeneca
- Moderna
- Novavax
- Johnson and Johnson
- I do not know which vaccine I received
- Other (please specify)

20. What was the date of your first vaccine dose? Please approximate if unsure.

| | Month | Day | Year |
|---|---|---|---|
| Date of first vaccine dose | | | |

21. For your second vaccine dose, which COVID-19 vaccine did you receive?

- Pfizer-BioNTech
- AstraZeneca
- Moderna
- Novavax
- Johnson and Johnson
- I do not know which vaccine I received
- Other (please specify)

22. What was the date of your second vaccine dose? Please approximate if unsure.

| | Month | Day | Year |
|---|---|---|---|
| Date of second vaccine dose | | | |

23. For your third vaccine dose, which COVID-19 vaccine did you receive?

- Pfizer-BioNTech
- AstraZeneca
- Moderna
- Novavax
- Johnson and Johnson
- I do not know which vaccine I received
- Other (please specify)

24. What was the date of your third vaccine dose? Please approximate if unsure.

| | Month | Day | Year |
|---|---|---|---|
| Date of third vaccine dose | | | |

25. For your fourth vaccine dose, which COVID-19 vaccine did you receive?

- ◯ Pfizer-BioNTech
- ◯ Moderna
- ◯ Johnson and Johnson
- ◯ Other (please specify)
- ◯ AstraZeneca
- ◯ Novavax
- ◯ I do not know which vaccine I received

26. What was the date of your fourth vaccine dose? Please approximate if unsure.

| | Month | Day | Year |
|---|---|---|---|
| Date of fourth vaccine dose | | | |

American Red Cross

## American Red Cross COVID-19 Follow-Up Study v4.01

**The next five sets of questions ask about persistent symptoms that you may have had since the start of the pandemic in March 2020, COVID-related or not.**

27. Since the start of the pandemic in March 2020, have you had any of these symptoms lasting over 4 consecutive weeks that you did not usually have before? Mark all that apply.

- ☐ Fatigue/weakness
- ☐ Headache
- ☐ Symptoms that get worse after physical or mental activities (post-exertional malaise)
- ☐ Problems sleeping
- ☐ Problems speaking or communicating
- ☐ Problems with balance/movement
- ☐ Tingling/numbness in any part of body
- ☐ Difficulty thinking or concentrating (sometimes referred to as "brain fog")
- ☐ Dizziness/lightheadedness/fainting
- ☐ Problems swallowing or chewing
- ☐ NONE OF THESE

American Red Cross

28. Please indicate which symptoms remain ongoing. Mark all that apply.

- ☐ Fatigue/weakness
- ☐ Headache
- ☐ Symptoms that get worse after physical or mental activities (post-exertional malaise)
- ☐ Problems sleeping
- ☐ Problems speaking or communicating
- ☐ Problems with balance/movement
- ☐ Tingling/numbness in any part of body
- ☐ Difficulty thinking or concentrating (sometimes referred to as "brain fog")
- ☐ Dizziness/lightheadedness/fainting
- ☐ Problems swallowing or chewing
- ☐ NONE OF THE ABOVE

29. Please indicate how long these symptoms lasted or have been ongoing.

| | Less than 2 months | 2 to 3 months | 4 to 5 months | 6 to 8 months | 9 to 12 months | More than 12 months |
|---|---|---|---|---|---|---|
| Fatigue/weakness | ○ | ○ | ○ | ○ | ○ | ○ |
| Headache | ○ | ○ | ○ | ○ | ○ | ○ |
| Symptoms that get worse after physical or mental activities (post-exertional malaise) | ○ | ○ | ○ | ○ | ○ | ○ |
| Problems sleeping | ○ | ○ | ○ | ○ | ○ | ○ |
| Problems speaking or communicating | ○ | ○ | ○ | ○ | ○ | ○ |
| Problems with balance/movement | ○ | ○ | ○ | ○ | ○ | ○ |
| Tingling/numbness in any part of body | ○ | ○ | ○ | ○ | ○ | ○ |
| Difficulty thinking or concentrating (sometimes referred to as "brain fog") | ○ | ○ | ○ | ○ | ○ | ○ |
| Dizziness/lightheadedness/fainting | ○ | ○ | ○ | ○ | ○ | ○ |
| Problems swallowing or chewing | ○ | ○ | ○ | ○ | ○ | ○ |

American Red Cross

## American Red Cross COVID-19 Follow-Up Study v4.01

30. Since the start of the pandemic in March 2020, have you had any of these symptoms lasting over 4 consecutive weeks that you did not usually have before? Mark all that apply.

- ☐ Appetite changes
- ☐ Constipation
- ☐ Diarrhea
- ☐ Nausea/vomiting
- ☐ Stomach pain
- ☐ NONE OF THESE

American Red Cross

## American Red Cross COVID-19 Follow-Up Study v4.01

31. Please indicate which symptoms remain ongoing. Mark all that apply.

- ☐ Appetite changes
- ☐ Constipation
- ☐ Diarrhea
- ☐ Nausea/vomiting
- ☐ Stomach pain
- ☐ NONE OF THE ABOVE

32. Please indicate how long these symptoms lasted or have been ongoing.

| | Less than 2 months | 2 to 3 months | 4 to 5 months | 6 to 8 months | 9 to 12 months | More than 12 months |
|---|---|---|---|---|---|---|
| Appetite changes | ○ | ○ | ○ | ○ | ○ | ○ |
| Constipation | ○ | ○ | ○ | ○ | ○ | ○ |
| Diarrhea | ○ | ○ | ○ | ○ | ○ | ○ |
| Nausea/vomiting | ○ | ○ | ○ | ○ | ○ | ○ |
| Stomach pain | ○ | ○ | ○ | ○ | ○ | ○ |

American Red Cross

## American Red Cross COVID-19 Follow-Up Study v4.01

33. Since the start of the pandemic in March 2020, have you had any of these symptoms lasting <u>over 4 consecutive weeks</u> that you did not usually have before? Mark all that apply.

- ☐ Congestion/runny nose
- ☐ Cough
- ☐ Shortness of breath
- ☐ Palpitations (heart racing or pounding)
- ☐ Sore throat
- ☐ Chest pain/pressure
- ☐ NONE OF THESE

American Red Cross

American Red Cross COVID-19 Follow-Up Study v4.01

34. Please indicate which symptoms remain ongoing. Mark all that apply.

- [ ] Congestion/runny nose
- [ ] Cough
- [ ] Shortness of breath
- [ ] Palpitations (heart racing or pounding)
- [ ] Sore throat
- [ ] Chest pain/pressure
- [ ] NONE OF THE ABOVE

35. Please indicate how long these symptoms lasted or have been ongoing.

| | Less than 2 months | 2 to 3 months | 4 to 5 months | 6 to 8 months | 9 to 12 months | More than 12 months |
|---|---|---|---|---|---|---|
| Congestion/runny nose | ○ | ○ | ○ | ○ | ○ | ○ |
| Cough | ○ | ○ | ○ | ○ | ○ | ○ |
| Shortness of breath | ○ | ○ | ○ | ○ | ○ | ○ |
| Palpitations (heart racing or pounding) | ○ | ○ | ○ | ○ | ○ | ○ |
| Sore throat | ○ | ○ | ○ | ○ | ○ | ○ |
| Chest pain/pressure | ○ | ○ | ○ | ○ | ○ | ○ |

American Red Cross

## American Red Cross COVID-19 Follow-Up Study v4.01

36. Since the start of the pandemic in March 2020, have you had any of these symptoms lasting over 4 consecutive weeks that you did not usually have before? Mark all that apply.

- [ ] Bruising/bleeding easily
- [ ] Change in menstruation cycle
- [ ] Fever or chills
- [ ] Hair loss
- [ ] Joint swelling
- [ ] Joint/muscle pain
- [ ] Skin changes
- [ ] Unintentional weight loss
- [ ] Change in taste
- [ ] Change in smell
- [ ] NONE OF THESE

## American Red Cross COVID-19 Follow-Up Study v4.01

37. Please indicate which symptoms remain ongoing. Mark all that apply.

- [ ] Bruising/bleeding easily
- [ ] Change in menstruation cycle
- [ ] Fever or chills
- [ ] Hair loss
- [ ] Joint swelling
- [ ] Joint/muscle pain
- [ ] Skin changes
- [ ] Unintentional weight loss
- [ ] Change in taste
- [ ] Change in smell
- [ ] NONE OF THE ABOVE

38. Please indicate how long these symptoms lasted or have been ongoing.

| | Less than 2 months | 2 to 3 months | 4 to 5 months | 6 to 8 months | 9 to 12 months | More than 12 months |
|---|---|---|---|---|---|---|
| Bruising/bleeding easily | ○ | ○ | ○ | ○ | ○ | ○ |
| Change in menstruation cycle | ○ | ○ | ○ | ○ | ○ | ○ |
| Fever or chills | ○ | ○ | ○ | ○ | ○ | ○ |
| Hair loss | ○ | ○ | ○ | ○ | ○ | ○ |
| Joint swelling | ○ | ○ | ○ | ○ | ○ | ○ |
| Joint/muscle pain | ○ | ○ | ○ | ○ | ○ | ○ |
| Skin changes | ○ | ○ | ○ | ○ | ○ | ○ |
| Unintentional weight loss | ○ | ○ | ○ | ○ | ○ | ○ |
| Change in taste | ○ | ○ | ○ | ○ | ○ | ○ |
| Change in smell | ○ | ○ | ○ | ○ | ○ | ○ |

American Red Cross

## American Red Cross COVID-19 Follow-Up Study v4.01

39. Since the start of the pandemic in March 2020, have you had any of these symptoms lasting over 4 consecutive weeks that you did not usually have before? Mark all that apply.

- ☐ Anxiety
- ☐ Depression
- ☐ Post-traumatic stress disorder (PTSD)
- ☐ Change in mood
- ☐ NONE OF THESE

American Red Cross

## American Red Cross COVID-19 Follow-Up Study v4.01

40. Please indicate which symptoms remain ongoing. Mark all that apply.

- ☐ Anxiety
- ☐ Depression
- ☐ Post-traumatic stress disorder (PTSD)
- ☐ Change in mood
- ☐ NONE OF THE ABOVE

41. Please indicate how long these symptoms lasted or have been ongoing.

| | Less than 2 months | 2 to 3 months | 4 to 5 months | 6 to 8 months | 9 to 12 months | More than 12 months |
|---|---|---|---|---|---|---|
| Anxiety | ○ | ○ | ○ | ○ | ○ | ○ |
| Depression | ○ | ○ | ○ | ○ | ○ | ○ |
| Post-traumatic stress disorder (PTSD) | ○ | ○ | ○ | ○ | ○ | ○ |
| Change in mood | ○ | ○ | ○ | ○ | ○ | ○ |

American Red Cross

## American Red Cross COVID-19 Follow-Up Study v4.01

42. For any of the symptoms lasting more than 4 consecutive weeks, did you ever see a doctor, nurse, or other health professional (including in-person and virtual visits)?

- ○ Yes
- ○ No
- ○ I don't know/am unsure

American Red Cross

American Red Cross COVID-19 Follow-Up Study v4.01

43. What kind(s) of doctor(s), nurse(s), or other health professional(s) did you see for the symptoms lasting more than 4 consecutive weeks? Mark all that apply.

- ☐ Primary care/general/family practitioner
- ☐ Cardiologist
- ☐ Dermatologist
- ☐ Mental health practitioner (counselor, psychologist, psychiatrist, etc)
- ☐ Other health professional (please specify)
- ☐ Neurologist
- ☐ Physical/occupational therapist
- ☐ Pulmonologist
- ☐ I don't know/am unsure

44. Do you attribute any of the symptom(s) lasting over 4 consecutive weeks to COVID-19 infection?

- ○ Yes
- ○ No
- ○ I don't know/am unsure

45. After your first COVID-19 infection, how long did it take to return to your usual health?

- Less than 1 month
- 1 to 2 months
- 3 to 5 months
- 6 to 8 months
- 9 to 12 months
- More than 12 months
- Have not returned to usual health
- I don't know/am unsure

American Red Cross

## American Red Cross COVID-19 Follow-Up Study v4.01

46. In the past 4 weeks, would you say that in general your health was:

- Excellent
- Very good
- Good
- Fair
- Poor

47. In the past 4 weeks, did poor physical or mental health keep you from doing your usual activities, such as self-care, work, or recreation?

- Yes, often
- Yes, sometimes
- No
- I don't know/am unsure

48. How does your current physical health compare with your physical health prior to the pandemic?

- Better than before the pandemic
- Worse than before the pandemic
- About the same
- I don't know/am unsure

49. How does your current mental health compare with your mental health prior to the pandemic?

- Better than before the pandemic
- Worse than before the pandemic
- About the same
- I don't know/am unsure

American Red Cross

## American Red Cross COVID-19 Follow-Up Study v4.01

50. Which of the following activities would you have been comfortable engaging in over the past month (from today) if permitted? Select all that apply.

- Domestic air travel
- International air travel
- Gathering indoors with friends or family who do not live with me
- Dining indoors at restaurants
- Going to indoor bars or nightclubs
- Going to indoor public places without a mask or face covering
- Attending in-person indoor religious services
- Attending outdoor events or concerts
- Attending indoor events, concerts, or shows
- NONE OF THESE

51. Looking forward, what activities do you anticipate being comfortable engaging in next month if permitted? Select all that apply.

- Domestic air travel
- International air travel
- Gathering indoors with friends or family who do not live with me
- Dining indoors at restaurants
- Going to indoor bars or nightclubs
- Going to indoor public places without a mask or face covering
- Attending in-person indoor religious services
- Attending outdoor events or concerts
- Attending indoor events, concerts, or shows
- NONE OF THESE

52. In the next month, if you anticipate engaging in new or more activities (such as travel, public events, gatherings with friends, indoor dining) compared to last month, what are the reasons? Select all that apply.

- ☐ I expect to be vaccinated or have been recently vaccinated
- ☐ I expect enough other people will be vaccinated
- ☐ I expect the number of new cases to decrease
- ☐ I expect facilities / restaurants / gyms to implement proper guidelines
- ☐ I expect policy changes at the local / state / national level
- ☐ I am willing to tolerate the risks for my personal well-being and mental health
- ☐ I don't expect to engage in new or more activities in the next month

53. In the next month, if you anticipate engaging in fewer activities (such as travel, public events, gatherings with friends, indoor dining) compared to last month, what are the reasons? Select all that apply.

- ☐ I am concerned about decreased immune protection against COVID-19
- ☐ I am concerned about SARS-CoV-2 variants
- ☐ I don't think enough other people are vaccinated
- ☐ I am concerned about current or future increase in cases
- ☐ I don't think facilities / restaurants / gyms are implementing proper restrictions
- ☐ I am concerned about low levels of mask use in public places
- ☐ I don't expect to engage in fewer activities next month

**Thank you for clicking through to learn more about our American Red Cross (ARC) survey!**

**You are eligible for this survey because you donated blood between June 15, 2020 and December 31, 2021, had one or more of these donations tested for antibodies to the virus that causes COVID-19 (SARS-CoV-2), and you completed a previous survey on your experience with COVID-19.**

**We appreciate your support of Red Cross research and are now inviting you to participate in an abbreviated follow-up survey on COVID-19. The purpose of the current survey is to better understand the association between donor antibodies to the COVID-19 virus and risk for subsequent infection and illness from SARS-CoV-2. Although you have previously completed one of our surveys on COVID-19, your participation in this survey is still valuable to help answer current scientific questions.**

**Please note that in this survey, we are not asking about your cumulative history with COVID-19 infection and vaccinations. Rather, we are asking about your experience since you last completed our survey, the date of which will be provided throughout the survey.**

**This research is being conducted in collaboration with the Centers for Disease Control and Prevention. Your participation is voluntary, and there will be no impact to your relationship with American Red Cross if you decide not to participate. Please note that information that can be linked to you will not be shared outside of ARC. If you agree to participate, please begin the survey below.**

**If you have questions or concerns, please contact us Monday through Friday, 8am to 4pm Central time at 855-563-7024.**

1. Since you last completed our survey on {{ contact.custom2 }}, have you been infected by SARS-CoV-2, the virus that causes COVID-19?
(*If you have been infected more than once, please answer for your earliest infection*).

- ◯ Yes, and my infection was confirmed by a diagnostic test (nose swab, throat swab, saliva test) or a healthcare provider (or both)
- ◯ Yes, but my infection was confirmed only by a blood test (antibody test, such as from Red Cross)
- ◯ Yes, I think I have been infected but it has not been confirmed by a test or diagnosis
- ◯ No
- ◯ I don't know/am unsure

2. Do you think you have been infected by the COVID-19 virus more than once between {{ contact.custom2 }} and today?

- ◯ Yes, twice
- ◯ No
- ◯ I don't know/am unsure

3. When were you first infected by the COVID-19 virus between {{ contact.custom2 }} and today?
Please approximate if unsure.

| | Month | Day | Year |
|---|---|---|---|
| Date of first COVID-19 infection between {{ contact.custom2 }} and today | | | |

4. When you were first infected by the COVID-19 virus between {{ contact.custom2 }} and today, did you have any symptoms?

- ◯ Yes
- ◯ No
- ◯ I don't know/am unsure

## American Red Cross COVID-19 Follow-Up Study_Short.FQ2

5. In the 2 weeks after your COVID-19 symptoms started, which of the following symptoms did you have? (This refers to the first time you were infected with the COVID-19 virus between {{ contact.custom2 }} and today). Mark all that apply.

- ☐ Fever
- ☐ Chills or rigors (shaking chills)
- ☐ Muscle aches
- ☐ Headache
- ☐ Sore throat
- ☐ Nausea or vomiting
- ☐ Diarrhea
- ☐ Fatigue
- ☐ Congestion or runny nose
- ☐ Cough
- ☐ Shortness of breath or difficulty breathing
- ☐ Loss of smell
- ☐ Loss of taste
- ☐ Confusion
- ☐ Persistent chest pain or chest pressure
- ☐ Pale, gray, or blue-colored skin, lips or nail beds
- ☐ Difficulty waking up or staying awake
- ☐ Other (please specify)

6. Were you hospitalized for COVID-19 for your first infection between {{ contact.custom2 }} and today? Choose the answer that best applies.

- ◯ Yes, I was hospitalized and admitted to the intensive care unit
- ◯ Yes, I was hospitalized but not admitted to the intensive care unit
- ◯ No, I was not hospitalized
- ◯ I don't know/am unsure

## American Red Cross COVID-19 Follow-Up Study_Short.FQ2

7. When do you think you were infected by the COVID-19 virus for the second time between {{ contact.custom2 }} and today?
Please approximate if unsure.

| | Month | Day | Year |
|---|---|---|---|
| Date of second COVID-19 infection between {{ contact.custom2 }} and today | | | |

8. Was your second infection from the COVID-19 virus between {{ contact.custom2 }} and today confirmed?

- ◯ Yes, and my infection was confirmed by a diagnostic test (nose swab, throat swab, saliva test) or a healthcare provider (or both)
- ◯ No. I think I have been infected but it has not been confirmed by a test or diagnosis.
- ◯ I don't know/am unsure

9. When you had your second infection with the COVID-19 virus between {{ contact.custom2 }} and today, did you have any symptoms?

- ◯ Yes
- ◯ No
- ◯ I don't know/am unsure

## American Red Cross COVID-19 Follow-Up Study_Short.FQ2

10. In the 2 weeks after your COVID-19 symptoms started, which of the following symptoms did you have? (This refers to the <u>second</u> time you were infected with the COVID-19 virus between {{ contact.custom2 }} and today). Mark all that apply.

- ☐ Fever
- ☐ Chills or rigors (shaking chills)
- ☐ Muscle aches
- ☐ Headache
- ☐ Sore throat
- ☐ Nausea or vomiting
- ☐ Diarrhea
- ☐ Fatigue
- ☐ Congestion or runny nose
- ☐ Cough
- ☐ Shortness of breath or difficulty breathing
- ☐ Loss of smell
- ☐ Loss of taste
- ☐ Confusion
- ☐ Persistent chest pain or chest pressure
- ☐ Pale, gray, or blue-colored skin, lips or nail beds
- ☐ Difficulty waking up or staying awake
- ☐ Other (please specify)

11. Were you hospitalized for your <u>second</u> COVID-19 infection between {{ contact.custom2 }} and today? Choose the answer that best applies.

- ◯ Yes, I was hospitalized and admitted to the intensive care unit
- ◯ Yes, I was hospitalized but not admitted to the intensive care unit
- ◯ No, I was not hospitalized
- ◯ I don't know/am unsure

## American Red Cross COVID-19 Follow-Up Study_Short.FQ2

12. Have you received any COVID-19 vaccines, including boosters between {{ contact.custom2 }} and today?
(*Please note you have previously provided information on vaccine doses, including boosters, that occurred <u>prior to</u> {{ contact.custom2 }} and we will connect the updated information with that you previously gave.)*

- ◯ Yes, one dose
- ◯ Yes, two doses
- ◯ No, but I plan to
- ◯ No, and I don't plan to
- ◯ I don't know/am unsure

## American Red Cross COVID-19 Follow-Up Study_Short.FQ2

13. For your first vaccine dose between {{ contact.custom2 }} and today, which COVID-19 vaccine did you receive?

- ◯ Pfizer-BioNTech
- ◯ Johnson and Johnson
- ◯ Moderna
- ◯ I do not know which vaccine I received
- ◯ Other (please specify)

[ ]

14. What was the date of your first vaccine dose between {{ contact.custom2 }} and today? Please approximate if unsure.

| | Month | Day | Year |
|---|---|---|---|
| Date of first vaccine dose between {{ contact.custom2 }} and today | [ ] | [ ] | [ ] |

15. For your second vaccine dose between {{ contact.custom2 }} and today, which COVID-19 vaccine did you receive?

- ◯ Pfizer-BioNTech
- ◯ Johnson and Johnson
- ◯ Moderna
- ◯ I do not know which vaccine I received
- ◯ Other (please specify)

[ ]

16. What was the date of your second vaccine dose between {{ contact.custom2 }} and today? Please approximate if unsure.

| | Month | Day | Year |
|---|---|---|---|
| Date of second vaccine dose between {{ contact.custom2 }} and today | [ ] | [ ] | [ ] |

**Understanding COVID-19 Baseline Survey**

**We are going to ask you two initial questions. Depending on your answers to these questions we may ask you additional questions to collect more information from you. We will also ask you about your general health today in the last part of the survey.**

**COVID-19 Infection**

1. Outside of COVID-19 antibody testing of your blood donation(s), have you ever had a positive COVID-19 result based on a swab test (a sample was collected from inside your nose or throat), or a saliva (spit) sample, or have you been diagnosed by a physician as having COVID-19?
   - Yes
   - No

**SARS-CoV-2 Vaccination**

2. Have you received a COVID-19 vaccine? This is also known as a SARS-CoV-2 vaccine.
   - Yes
   - No

If 1. = Yes, go to 3.

If 2. = Yes, go to 13.

If 1 = No and 2 = No, go to 19.

**COVID-19 Testing and Symptom History**

**Since you indicated you have had a positive test for COVID-19, we are going to ask you more questions about COVID-19 testing and symptoms you may have had.**

3. When did you first have a positive COVID-19 test? [Select Date: Month_Day_Year]

4. Which type of COVID-19 test did you have?
   - Virus Test. If you have a laboratory report it may call this test a Nucleic Acid Amplification Test (NAAT) or a Polymerase Chain Reaction (PCR) Test.

   - Antigen Test

     If selected: Do you know if this was a rapid test where the results were available to you within 20 minutes?

       - Yes
       - No
       - Unsure

   - Antibody Test. Please don’t include testing done from your blood donation(s) when answering.

     If selected: Do you know if this was a rapid test where the results were available to you within 20 minutes?

       - Yes
       - No
       - Unsure
   - Unsure

5. Have you had more than one positive COVID-19 test?
   - Yes Go to 6.
   - No Go to Q8
   - Unsure Go to Q8

6. When did you have your second positive COVID-19 test? [Select Date: Month_Day_Year]

7. Which type of COVID-19 test did you have?
   - Nucleic Acid Amplification Test (NAAT). This is also known as a Polymerase Chain Reaction (PCR) Test

   - Antigen Test

     If selected: Do you know if this was a rapid test where the results were available to you within 20 minutes?

       - Yes

- No
- Unsure

- Antibody

If selected: Do you know if this was a rapid test where the results were available to you within 20 minutes?

- Yes
- No
- Unsure

- Unsure

8. Did you have any symptoms before or after (either of) your positive COVID-19 test(s)?

- Yes
- No
- Unsure

9. a. Select the symptoms that you had around the time of your FIRST (or only) positive COVID-19 test. Please do not include possible reactions following vaccination when answering these questions.

b. Select the symptoms that you had around the time of your SECOND positive COVID-19 test. Please do not include possible reactions following vaccination when answering these questions.

| | Experienced? | | | If Yes, how many episodes did you have? A symptom episode lasts several days in a row. | How many days did the average episode last? |
|---|---|---|---|---|---|
| | Yes | No | Unsure | | |
| Cough | | | | | |
| Shortness of breath or difficulty breathing | | | | | |
| Sore throat | | | | | |
| Runny nose | | | | | |
| Loss of taste or smell | | | | | |
| Fever | | | | | |
| Headache | | | | | |
| Muscle or body aches | | | | | |
| Nausea, vomiting, or diarrhea | | | | | |
| Extreme fatigue | | | | | |
| Trouble concentrating, also known as “brain fog” | | | | | |
| Problems with memory | | | | | |

10. Did you seek healthcare at any of these because of your illness?
    a. Hospital/ER
    b. Urgent care
    c. Physician's office or clinic
    d. Telehealth
    e. Other
    f. Did not seek healthcare

11. Were you hospitalized because of your illness?
    a. Yes
    b. No

12. a. Would you describe yourself as having had 'long COVID', that is, did you experience symptoms that lasted for at least four weeks after being first infected that were not explained by something else?
    - Yes
    - No
    - Unsure

    If yes:

    b. Are these 'long COVID' symptoms still occurring today?
    - Yes
    - No
    - Unsure

**SARS-CoV-2 Vaccination Details**

**You indicated you have been vaccinated for COVID-19. To answer the next questions, it will be very helpful if you have your vaccination card in front of you. This is the card that was given to you by the person who gave you the vaccine.**

13. Please refer to your vaccination card to provide the following:

| | Received | | If received, please select Vaccine Name | If Other: Enter name of other vaccine | Date Received: Month | Date Received: Day | Date Received: Year |
|---|---|---|---|---|---|---|---|
| | Yes | No | | | | | |
| COVID-19 Vaccine First Dose | | | | | | | |
| COVID-19 Vaccine Second Dose | | | | | | | |
| Dose of the Same Vaccine or Booster | | | | | | | |

14. What motivated you to get vaccinated? Select all that apply.

- ◻ Protect my health
- ◻ Protect health of family/friends
- ◻ Protect health of co-workers/community
- ◻ To get back to work/school
- ◻ To resume social activities
- ◻ To resume travel
- ◻ Because others encouraged me to get vaccinated
- ◻ Not sure
- ◻ Other

15. Did you have a reaction following any of your COVID-19 vaccinations?
   - ○ Yes
   - ○ No
   - ○ Unsure

16. Select the reaction you had after your FIRST COVID-19 vaccination:
    Select the reaction you had after your SECOND COVID-19 vaccination:
    Select the reaction you had after your OTHER COVID-19 vaccination or BOOSTER:

| | Experienced? | | | If Yes, for how long? | | |
|---|---|---|---|---|---|---|
| | Yes | No | Unsure | Less than 1 day | 1 to 3 days | 4 days or more |
| Soreness, redness, or swelling at injection site | | | | | | |
| Fever or chills | | | | | | |
| Muscle pain or any other body aches | | | | | | |
| Fatigue | | | | | | |
| Nausea | | | | | | |

**Opinions on Vaccination**

**The next questions cover your opinions about vaccination. There are no right answers, please tell us what you think:**

**If 2 = Yes, ask 17, 18, and 22:**

17. How concerned were you about getting a COVID-19 vaccine?
    - Not at all concerned
    - A little concerned
    - Moderately concerned
    - Very concerned
    - Not sure

18. How easy was it to get a COVID-19 vaccine for yourself?
    - Very easy
    - Somewhat easy
    - Somewhat difficult
    - Very difficult
    - Not sure

**If 2 = No, ask 19-22:**

19. How concerned are you about getting a COVID-19 vaccine?

    - Not at all concerned
    - A little concerned
    - Moderately concerned
    - Very concerned
    - Not sure

20. What makes you not want to get a COVID-19 vaccine? Select all that apply.

    - ☐ Unsure the vaccines are safe.
    - ☐ Personal, cultural, or religious beliefs against vaccination.
    - ☐ I am young and do not need to worry about being vaccinated.
    - ☐ I am worried about being exposed to SARS-CoV-2 when I am at the vaccination location.
    - ☐ I can’t go on my own (I have a physical or other limitation) or don’t have transportation.
    - ☐ I don’t know where to go to get vaccinated.
    - ☐ I’m not eligible to get a COVID-19 vaccine or have a medical reason that makes me ineligible to get vaccinated (e.g., I have had a severe allergy to vaccines in the past).
    - ☐ The vaccination clinic is too far away or the hours of operation are inconvenient.
    - ☐ The waiting time is too long. It is difficult to find or make an appointment.
    - ☐ I am too busy to get vaccinated.
    - ☐ It is difficult to arrange for childcare.
    - ☐ I don’t have time off work.
    - ☐ Other
    - ☐ Not sure

21. How easy would it be to get a COVID-19 vaccine for yourself?

    - Very easy
    - Somewhat easy
    - Somewhat difficult
    - Very difficult
    - Not sure

22. How likely are you to recommend getting the COVID-19 vaccine to others?

    - Not at all likely
    - Somewhat likely
    - Extremely likely
    - Not sure

**Medical History Information**

**The final section of the survey will ask you about other parts of your medical history. These data will help us to understand whether other health-related topics influence the chance of getting SARS-CoV-2 or COVID-19.**

23. On the scale below showing 0 to 10, please rate your current health today by dragging or touching the line below. 0 is the worst possible health you can imagine and 10 is the best possible health you can imagine.

0 1 2 3 4 5 6 7 8 9 10

24. Have you had, or do you currently have any of the following health conditions? Check all that apply.
    - ☐ Asthma
    - ☐ Other respiratory disease
    - ☐ Heart disease or other cardiovascular disease
    - ☐ High blood pressure
    - ☐ Diabetes
    - ☐ Any kind of immune system disorder
    - ☐ Kidney disease
    - ☐ Liver disease
    - ☐ Neurological disease
    - ☐ Cancer
    - ☐ None of the above

25. During the past 30 days, have you smoked part or all of a cigarette?
    - ○ Yes
    - ○ No

**If 25=Yes, ask 25a.**

25a. On average, during the past 30 days, how many cigarettes did you smoke per day?
- ○ Zero
- ○ More than zero, but less than one cigarette
- ○ 1 cigarette
- ○ 2 to 5 cigarettes
- ○ 6 to 15 cigarettes
- ○ 16 to 25 cigarettes
- ○ 26 to 35 cigarettes
- ○ More than 35 cigarettes
- ○ Not sure

**Submission Confirmation Page**

**If consented:**
You have reached the end of the survey.

Thank you for taking the time to provide these answers to us. We truly appreciate your donations and willingness to be a part of research conducted at Vitalant Research Institute. We will send you a survey about every three months to update your information as part of your participation in this research. You may opt out at any time by declining the next survey invitation.

**If did not consent:**
You have reached the end of the survey.

**END**

# Understanding COVID-19 Follow-up Survey

**We are going to ask you two initial questions. The answers to these may have changed since your last survey on [DATE]. Depending on your answers to these questions we may ask you additional questions to collect more information from you. We will also ask you about your general health today in the last part of the survey.**

## COVID-19 Infection

1. Outside of COVID-19 antibody testing of your blood donation(s), **since your last survey** have you had a positive COVID-19 result based on a swab test (a sample was collected from your inside your nose or throat), a saliva (spit) sample, or been diagnosed by a physician as having COVID-19?

   - o Yes
   - o No

## SARS-CoV-2 Vaccination

2. **Since your last survey** have you received any new or additional doses of a COVID-19 vaccine? This is also known as a SARS-CoV-2 vaccine.

   - o Yes
   - o No

If 1. = Yes, go to 3.

If 2. = Yes, go to 14.

If 1 = No and 2 = No, go to 18.

**COVID-19 Testing and Symptom History**

**Since you indicated you have had a positive test or physician diagnosis for COVID-19 since your last survey on [DATE], we are going to ask you more questions about COVID-19 testing and symptoms you may have had.**

3. How many positive COVID-19 tests or physician diagnoses have you had since your last survey?
   - o 1
   - o 2
   - o 3
   - o 4
   - o 5 or more

The questions below are in regard to your FIRST (or only) positive COVID-19 test or physician diagnosis since your last survey on [DATE].

If more than one in Q3:
The questions below are in regard to your [Nth] positive COVID-19 test or physician diagnosis since your last survey.

4a. When did you have your first positive COVID-19 test or physician diagnosis since your last survey?
[Select Date: Month_Day_Year]

If more than one in Q3:
4b-n. When did you have your [nth] positive COVID-19 test or physician diagnosis?

5a. Which type of COVID-19 test did you have?

If more than one:
5b-n. Which type of COVID-19 test did you have?

- o Virus Test. If you have a laboratory report it may call this test a Nucleic Acid Amplification Test (NAAT) or a Polymerase Chain Reaction (PCR) Test.
- o Antigen Test

  If selected: Do you know if this was a rapid test where the results were available to you within 20 minutes?

  - o Yes
  - o No
  - o Unsure

- Antibody Test. Please don't include testing done from your blood donation(s) when answering.

  If selected: Do you know if this was a rapid test where the results were available to you within 20 minutes?

  - Yes
  - No
  - Unsure

- Diagnosed by physician

- Unsure

6a. Did you have any symptoms around the time of your positive COVID-19 test or physician diagnosis?

If more than one in Q3:
6b-n. Did you have any symptoms around the time of your positive COVID-19 test or physician diagnosis?

- Yes
- No
- Unsure

  Yes: Go to Q7

  No: Go to 15

7a. Select the symptoms that you had around the time of your positive COVID-19 test or physician diagnosis. Please do not include possible reactions following vaccination when answering these questions.

If more than one in Q3:
7b-n. Select the symptoms that you had around the time of your positive COVID-19 test or physician diagnosis. Please do not include possible reactions following vaccination when answering these questions.

| | Experienced? | | | If Yes, how many episodes did you have? A symptom episode lasts several days in a row. | How many days did the average episode last? |
|---|---|---|---|---|---|
| | Yes | No | Unsure | | |
| Cough | | | | | |
| Shortness of breath or difficulty breathing | | | | | |
| Sore throat | | | | | |
| Congestion or runny nose | | | | | |
| Loss of taste or smell | | | | | |
| Fever | | | | | |
| Headache | | | | | |
| Muscle or body aches | | | | | |
| Nausea, vomiting, or diarrhea | | | | | |
| Extreme fatigue | | | | | |
| Trouble concentrating, also known as “brain fog” | | | | | |
| Problems with memory | | | | | |
| Other: [In-line open text box] | | | | | |

8a. Did you seek healthcare at any of these because of this illness?

If more than one in Q3:
8b-n. Did you seek healthcare at any of these because of this illness?

- ☐ Hospital/ER
- ☐ Urgent care
- ☐ Physician’s office or clinic
- ☐ Telehealth
- ☐ Other: [In-line open text box]
- ☐ Did not seek healthcare

9a. Were you hospitalized because of this illness?

If more than one in Q3:
9b-n. Were you hospitalized because of this illness?

- ○ Yes
- ○ No

The next questions ask about potential long COVID symptoms. Also known as post-acute sequelae of SARS-CoV-2 infection (PASC), long COVID refers to a range of symptoms that can last for months after first being infected with SARS-CoV-2. They can even first appear weeks after the infection has resolved. Long COVID can happen to anyone infected with SARS-CoV-2, even if the illness was mild or entirely asymptomatic.

10. Did you experience symptoms that lasted for at least four weeks any time after being first infected with SARS-CoV-2 that were not explained by something else?
    - o Yes
    - o No

11. Please mark any symptoms lasting 4 or more weeks that you may have had, indicating how long these symptoms lasted and if they are still ongoing.

| | Experienced? | | | If Yes, for how long did symptoms last? [<2 months; 2-3 months; 4-5 months; 6-8 months; 9-12 months; More than 12 months] | If Yes, is this symptom still ongoing? | | |
|---|---|---|---|---|---|---|---|
| | Yes | No | Unsure | | Yes | No | Unsure |
| Fatigue/weakness | | | | | | | |
| Headache | | | | | | | |
| Symptoms that get worse after physical or mental activities (post-exertional malaise) | | | | | | | |
| Problems sleeping | | | | | | | |
| Problems speaking or communicating | | | | | | | |
| Problems with balance/movement | | | | | | | |
| Tingling/numbness in any part of body | | | | | | | |
| Difficulty thinking or concentrating (sometimes referred to as "brain fog") | | | | | | | |
| Dizziness/light headedness/fainting | | | | | | | |
| Problems swallowing or chewing | | | | | | | |

| | | | | | |
|---|---|---|---|---|---|
| Appetite changes | | | | | |
| Constipation | | | | | |
| Diarrhea | | | | | |
| Nausea/vomiting | | | | | |
| Stomach pain | | | | | |
| Congestion/runny nose | | | | | |
| Cough | | | | | |
| Shortness of breath | | | | | |
| Palpitations (heart racing or pounding) | | | | | |
| Sore throat | | | | | |
| Chest pain/pressure | | | | | |
| Bruising/bleeding easily | | | | | |
| Change in menstruation cycle | | | | | |
| Fever or chills | | | | | |
| Hair loss | | | | | |
| Joint swelling | | | | | |
| Joint/muscle pain | | | | | |
| Skin changes | | | | | |
| Unintentional weight loss | | | | | |
| Change in taste | | | | | |
| Change in smell | | | | | |
| Anxiety | | | | | |
| Depression | | | | | |
| Post-traumatic stress disorder (PTSD) | | | | | |
| Change in mood | | | | | |
| Other: [In-line open text box] | | | | | |

12. a. For any of the symptoms lasting more than 4 consecutive weeks, did you ever see a doctor, nurse, or other health professional (including in-person and virtual visits)?
    - o Yes
    - o No
    - o I don't know/unsure

    b. If yes: What kind(s) of doctor(s), nurse(s) or other health professional(s) did you see for the symptoms(s) lasting more than 4 consecutive weeks (check all that apply)?

    - o Primary care/general/family practitioner
    - o Cardiologist
    - o Dermatologist

- Mental health practitioner (counselor, psychologist, psychiatrist, etc.)
- Neurologist
- Physical/occupational therapist
- Pulmonologist
- I don't know/am unsure
- Other health professional (please specify): [In-line open text box]

13. a. Would you describe yourself as having 'long COVID', that is, do you attribute any of the symptom(s) lasting more than 4 weeks to COVID-19 infection?
    - Yes
    - No
    - Unsure

    b. If yes: Are these 'long COVID' symptoms still occurring today?

    - Yes
    - No
    - Unsure

**SARS-CoV-2 Vaccination Details**

**You indicated you have been vaccinated for COVID-19 since your last survey on [DATE]. To answer the next questions, it will be very helpful if you have your vaccination card in front of you. This is the card that was given to you by the person who gave you the vaccine.**

14. Please refer to your vaccination card to provide the following for any vaccines or boosters received after you completed your last survey.

| | What vaccine dose was this? Drop-down:<br>• First COVID-19 Vaccine Dose<br>• Second COVID-19 Vaccine Dose<br>• Third COVID-19 Vaccine Dose or Booster<br>• Fourth COVID-19 Vaccine Dose or Booster<br>• Nth COVID-19 Vaccine Dose or Booster | Select Vaccine Name Drop-down:<br>• Pfizer-BioNTech<br>• Moderna<br>• Janssen, Johnson & Johnson, or J&J<br>• Novavax<br>• AstraZeneca-Oxford<br>• Other | If Other: | Date Received: Month | Date Received: Day | Date Received: Year |
|---|---|---|---|---|---|---|
| Additional COVID-19 Vaccination 1 | | | | | | |
| Additional COVID-19 Vaccination 2 | | | | | | |
| Additional COVID-19 Vaccination 3 | | | | | | |
| … | | | | | | |
| Additional COVID Vaccination N | | | | | | |

15. a. What motivated you to get vaccinated? Select all that apply.

- ▫ Protect my health
- ▫ Protect health of family/friends

- ◻ Protect health of co-workers/community
- ◻ To get back to work/school
- ◻ To resume social activities
- ◻ To resume travel
- ◻ Because others encouraged me to get vaccinated
- ◻ Not sure
- ◻ Other: [In-line open text box]

16. Did you have a reaction following any of your COVID-19 vaccinations?
    - o Yes
    - o No
    - o Unsure

17. a. Select the reaction you had after your FIRST additional COVID-19 vaccination:
    b. Select the reaction you had after your SECOND additional COVID-19 vaccination:
    c. Select the reaction you had after your THIRD additional COVID-19 vaccination:
    …
    n. Select the reaction you had after your Nth additional COVID-19 vaccination:

| | Experienced? | | | If Yes, for how long? | | |
|---|---|---|---|---|---|---|
| | Yes | No | Unsure | Less than 1 day | 1 to 3 days | 4 days or more |
| Soreness, redness, or swelling at injection site | | | | | | |
| Fever or chills | | | | | | |
| Muscle pain or any other body aches | | | | | | |
| Fatigue | | | | | | |
| Nausea | | | | | | |
| Swollen lymph nodes in armpit or near collar bone on the side of body where vaccine was administered | | | | | | |
| Other: [In-line open text box] | | | | | | |

**Medical History Information**

**The final question of the survey asks about your general health today. These data will help us to understand whether other health-related topics influence the chance of getting SARS-CoV-2 or COVID-19.**

18. On the scale below showing 0 to 10, please rate your current health today by dragging or touching the line below. 0 is the worst possible health you can imagine and 10 is the best possible health you can imagine.

0 1 2 3 4 5 6 7 8 9 10

**If consented:**
You have reached the end of the survey.

Thank you for taking the time to provide these answers to us. We truly appreciate your donations and willingness to be a part of research conducted at Vitalant Research Institute. We will send you a survey about every three months to update your information as part of your participation in this research. You may opt out at any time by declining the next survey invitation.

**If did not consent:**
You have reached the end of the survey.

**END**

## American Red Cross COVID-19 Follow-Up Study v4.01

**Thank you for clicking through to learn more about our American Red Cross (ARC) survey!**

**You are eligible for this survey because you donated between June 15, 2020 and December 31, 2021.**

**We are inviting you to participate in a follow-up survey on COVID-19. The purpose of the current survey is to better understand the impact of the pandemic and COVID-19 infection on donor health. If you have previously completed one of our surveys on COVID-19, your participation in this survey is still valuable to help answer new scientific questions.**

**This research is being conducted in collaboration with the Centers for Disease Control and Prevention. Your participation is voluntary, and there will be no impact to your relationship with American Red Cross if you decide not to participate. Please note that information that can be linked to you will not be shared outside of ARC. If you agree to participate, please begin the survey below.**

**If you have questions or concerns, please contact us Monday through Friday, 10am to 6pm Eastern time at 866-628-9875.**

1. Since March 2020, have you been infected by SARS-CoV-2, the virus that causes COVID-19?
(*If you have been infected more than once, please answer for your earliest infection*).

- ◯ Yes, and my infection was confirmed by a diagnostic test (nose swab, throat swab, saliva test) or a healthcare provider (or both)
- ◯ Yes, but my infection was confirmed only by a blood test (antibody test, such as from Red Cross)
- ◯ Yes, I think I have been infected but it has not been confirmed by a test or diagnosis
- ◯ No
- ◯ I don't know/am unsure

## American Red Cross COVID-19 Follow-Up Study v4.01

2. Do you think you have been infected by the COVID-19 virus more than once?

- Yes, twice
- Yes, three times
- No
- I don't know/am unsure

3. When were you first infected by the COVID-19 virus?
Please approximate if unsure.

| | Month | Day | Year |
|---|---|---|---|
| Date of first COVID-19 infection | | | |

4. When you were first infected by the COVID-19 virus, did you have any symptoms?

- Yes
- No
- I don't know/am unsure

American Red Cross

## American Red Cross COVID-19 Follow-Up Study v4.01

5. In the 2 weeks after your COVID-19 symptoms started, which of the following symptoms did you have? (This refers to the first time you were infected with the COVID-19 virus). Mark all that apply.

- Fever
- Chills or rigors (shaking chills)
- Muscle aches
- Headache
- Sore throat
- Nausea or vomiting
- Diarrhea
- Fatigue
- Congestion or runny nose
- Cough
- Shortness of breath or difficulty breathing
- Loss of smell
- Loss of taste
- Confusion
- Persistent chest pain or chest pressure
- Pale, gray, or blue-colored skin, lips or nail beds
- Difficulty waking up or staying awake
- Other (please specify)

6. Were you hospitalized for COVID-19 for your <u>first</u> infection? Choose the answer that best applies.

- Yes, I was hospitalized and admitted to the intensive care unit
- Yes, I was hospitalized but not admitted to the intensive care unit
- No, I was not hospitalized
- I don't know/am unsure

American Red Cross

## American Red Cross COVID-19 Follow-Up Study v4.01

7. When do you think you were infected by the COVID-19 virus for the <u>second</u> time?
Please approximate if unsure.

| | Month | Day | Year |
|---|---|---|---|
| Date of <u>second</u> COVID-19 infection | | | |

8. Was your <u>second</u> infection from the COVID-19 virus confirmed?

- Yes, and my infection was confirmed by a diagnostic test (nose swab, throat swab, saliva test) or a healthcare provider (or both)
- No. I think I have been infected but it has not been confirmed by a test or diagnosis.
- I don't know/am unsure

9. When you had your second infection with the COVID-19 virus, did you have any symptoms?

- Yes
- No
- I don't know/am unsure

American Red Cross

## American Red Cross COVID-19 Follow-Up Study v4.01

10. In the 2 weeks after your COVID-19 symptoms started, which of the following symptoms did you have? (This refers to the second time you were infected with the COVID-19 virus). Mark all that apply.

- ☐ Fever
- ☐ Chills or rigors (shaking chills)
- ☐ Muscle aches
- ☐ Headache
- ☐ Sore throat
- ☐ Nausea or vomiting
- ☐ Diarrhea
- ☐ Fatigue
- ☐ Congestion or runny nose
- ☐ Cough
- ☐ Shortness of breath or difficulty breathing
- ☐ Loss of smell
- ☐ Loss of taste
- ☐ Confusion
- ☐ Persistent chest pain or chest pressure
- ☐ Pale, gray, or blue-colored skin, lips or nail beds
- ☐ Difficulty waking up or staying awake
- ☐ Other (please specify)

11. Were you hospitalized for your second COVID-19 infection? Choose the answer that best applies.

- ○ Yes, I was hospitalized and admitted to the intensive care unit
- ○ Yes, I was hospitalized but not admitted to the intensive care unit
- ○ No, I was not hospitalized
- ○ I don't know/am unsure

American Red Cross

## American Red Cross COVID-19 Follow-Up Study v4.01

12. When do you think you were infected by the COVID-19 virus for the third time? Please approximate if unsure.

| | Month | Day | Year |
|---|---|---|---|
| Date of third COVID-19 infection | | | |

13. Was your third infection from the COVID-19 virus confirmed?

- ○ Yes, and my infection was confirmed by a diagnostic test (nose swab, throat swab, saliva test) or a healthcare provider (or both)
- ○ No. I think I have been infected but it has not been confirmed by a test or diagnosis.
- ○ I don't know/am unsure

14. When you had your third infection with the COVID-19 virus, did you have any symptoms?

- ◯ Yes
- ◯ No
- ◯ I don't know/am unsure

## American Red Cross COVID-19 Follow-Up Study v4.01

15. In the 2 weeks after your COVID-19 symptoms started, which of the following symptoms did you have? (This refers to the third time you were infected with the COVID-19 virus). Mark all that apply.

- ☐ Fever
- ☐ Chills or rigors (shaking chills)
- ☐ Muscle aches
- ☐ Headache
- ☐ Sore throat
- ☐ Nausea or vomiting
- ☐ Diarrhea
- ☐ Fatigue
- ☐ Congestion or runny nose
- ☐ Cough
- ☐ Shortness of breath or difficulty breathing
- ☐ Loss of smell
- ☐ Loss of taste
- ☐ Confusion
- ☐ Persistent chest pain or chest pressure
- ☐ Pale, gray, or blue-colored skin, lips or nail beds
- ☐ Difficulty waking up or staying awake
- ☐ Other (please specify)

16. Were you hospitalized for your third COVID-19 infection? Choose the answer that best applies.

- ◯ Yes, I was hospitalized and admitted to the intensive care unit
- ◯ Yes, I was hospitalized but not admitted to the intensive care unit
- ◯ No, I was not hospitalized
- ◯ I don't know/am unsure

## American Red Cross COVID-19 Follow-Up Study v4.01

17. Prior to the COVID-19 pandemic (March 2020), did a health care professional ever tell you that you had any of these chronic health conditions? Mark all that apply.

- ☐ Chronic pain
- ☐ Anxiety
- ☐ Depression
- ☐ Chronic headaches or migraines
- ☐ Stroke
- ☐ Kidney disease
- ☐ Liver disease
- ☐ Heart disease or other cardiovascular disease
- ☐ Lung disease/asthma
- ☐ High blood pressure
- ☐ Diabetes
- ☐ Any immune system disorder
- ☐ Cancer
- ☐ NONE OF THESE

American Red Cross

## American Red Cross COVID-19 Follow-Up Study v4.01

18. Have you received any COVID-19 vaccines, including boosters?

- ◯ Yes, one dose
- ◯ Yes, two doses
- ◯ Yes, three doses
- ◯ Yes, four doses
- ◯ No, but I plan to
- ◯ No, and I don't plan to
- ◯ I don't know/am unsure

American Red Cross

## American Red Cross COVID-19 Follow-Up Study v4.01

19. For your <u>first</u> vaccine dose, which COVID-19 vaccine did you receive?

- ◯ Pfizer-BioNTech
- ◯ Moderna
- ◯ Johnson and Johnson
- ◯ Other (please specify)
- ◯ AstraZeneca
- ◯ Novavax
- ◯ I do not know which vaccine I received

20. What was the date of your <u>first</u> vaccine dose? Please approximate if unsure.

| | Month | Day | Year |
|---|---|---|---|
| Date of <u>first</u> vaccine dose | | | |

21. For your <u>second</u> vaccine dose, which COVID-19 vaccine did you receive?

- ◯ Pfizer-BioNTech
- ◯ Moderna
- ◯ Johnson and Johnson
- ◯ Other (please specify)
- ◯ AstraZeneca
- ◯ Novavax
- ◯ I do not know which vaccine I received

22. What was the date of your <u>second</u> vaccine dose? Please approximate if unsure.

| | Month | Day | Year |
|---|---|---|---|
| Date of <u>second</u> vaccine dose | | | |

23. For your <u>third</u> vaccine dose, which COVID-19 vaccine did you receive?

- ◯ Pfizer-BioNTech
- ◯ Moderna
- ◯ Johnson and Johnson
- ◯ Other (please specify)
- ◯ AstraZeneca
- ◯ Novavax
- ◯ I do not know which vaccine I received

24. What was the date of your <u>third</u> vaccine dose? Please approximate if unsure.

| | Month | Day | Year |
|---|---|---|---|
| Date of <u>third</u> vaccine dose | | | |

25. For your fourth vaccine dose, which COVID-19 vaccine did you receive?

- ◯ Pfizer-BioNTech
- ◯ Moderna
- ◯ Johnson and Johnson
- ◯ Other (please specify)
- ◯ AstraZeneca
- ◯ Novavax
- ◯ I do not know which vaccine I received

[ ]

26. What was the date of your fourth vaccine dose? Please approximate if unsure.

| | Month | Day | Year |
|---|---|---|---|
| Date of fourth vaccine dose | | | |

American Red Cross

## American Red Cross COVID-19 Follow-Up Study v4.01

**The next five sets of questions ask about persistent symptoms that you may have had since the start of the pandemic in March 2020, COVID-related or not.**

27. Since the start of the pandemic in March 2020, have you had any of these symptoms lasting over 4 consecutive weeks that you did not usually have before? Mark all that apply.

- ☐ Fatigue/weakness
- ☐ Headache
- ☐ Symptoms that get worse after physical or mental activities (post-exertional malaise)
- ☐ Problems sleeping
- ☐ Problems speaking or communicating
- ☐ Problems with balance/movement
- ☐ Tingling/numbness in any part of body
- ☐ Difficulty thinking or concentrating (sometimes referred to as "brain fog")
- ☐ Dizziness/lightheadedness/fainting
- ☐ Problems swallowing or chewing
- ☐ NONE OF THESE

American Red Cross

28. Please indicate which symptoms remain ongoing. Mark all that apply.

- ☐ Fatigue/weakness
- ☐ Headache
- ☐ Symptoms that get worse after physical or mental activities (post-exertional malaise)
- ☐ Problems sleeping
- ☐ Problems speaking or communicating
- ☐ Problems with balance/movement
- ☐ Tingling/numbness in any part of body
- ☐ Difficulty thinking or concentrating (sometimes referred to as "brain fog")
- ☐ Dizziness/lightheadedness/fainting
- ☐ Problems swallowing or chewing
- ☐ NONE OF THE ABOVE

29. Please indicate how long these symptoms lasted or have been ongoing.

| | Less than 2 months | 2 to 3 months | 4 to 5 months | 6 to 8 months | 9 to 12 months | More than 12 months |
|---|---|---|---|---|---|---|
| Fatigue/weakness | ○ | ○ | ○ | ○ | ○ | ○ |
| Headache | ○ | ○ | ○ | ○ | ○ | ○ |
| Symptoms that get worse after physical or mental activities (post-exertional malaise) | ○ | ○ | ○ | ○ | ○ | ○ |
| Problems sleeping | ○ | ○ | ○ | ○ | ○ | ○ |
| Problems speaking or communicating | ○ | ○ | ○ | ○ | ○ | ○ |
| Problems with balance/movement | ○ | ○ | ○ | ○ | ○ | ○ |
| Tingling/numbness in any part of body | ○ | ○ | ○ | ○ | ○ | ○ |
| Difficulty thinking or concentrating (sometimes referred to as "brain fog") | ○ | ○ | ○ | ○ | ○ | ○ |
| Dizziness/lightheadedness/fainting | ○ | ○ | ○ | ○ | ○ | ○ |
| Problems swallowing or chewing | ○ | ○ | ○ | ○ | ○ | ○ |

## American Red Cross COVID-19 Follow-Up Study v4.01

30. Since the start of the pandemic in March 2020, have you had any of these symptoms lasting over 4 consecutive weeks that you did not usually have before? Mark all that apply.

- ☐ Appetite changes
- ☐ Constipation
- ☐ Diarrhea
- ☐ Nausea/vomiting
- ☐ Stomach pain
- ☐ NONE OF THESE

## American Red Cross COVID-19 Follow-Up Study v4.01

31. Please indicate which symptoms remain ongoing. Mark all that apply.

- ☐ Appetite changes
- ☐ Constipation
- ☐ Diarrhea
- ☐ Nausea/vomiting
- ☐ Stomach pain
- ☐ NONE OF THE ABOVE

32. Please indicate how long these symptoms lasted or have been ongoing.

| | Less than 2 months | 2 to 3 months | 4 to 5 months | 6 to 8 months | 9 to 12 months | More than 12 months |
|---|---|---|---|---|---|---|
| Appetite changes | ◯ | ◯ | ◯ | ◯ | ◯ | ◯ |
| Constipation | ◯ | ◯ | ◯ | ◯ | ◯ | ◯ |
| Diarrhea | ◯ | ◯ | ◯ | ◯ | ◯ | ◯ |
| Nausea/vomiting | ◯ | ◯ | ◯ | ◯ | ◯ | ◯ |
| Stomach pain | ◯ | ◯ | ◯ | ◯ | ◯ | ◯ |

American Red Cross

American Red Cross COVID-19 Follow-Up Study v4.01

33. Since the start of the pandemic in March 2020, have you had any of these symptoms lasting over 4 consecutive weeks that you did not usually have before? Mark all that apply.

- ☐ Congestion/runny nose
- ☐ Cough
- ☐ Shortness of breath
- ☐ Palpitations (heart racing or pounding)
- ☐ Sore throat
- ☐ Chest pain/pressure
- ☐ NONE OF THESE

American Red Cross

American Red Cross COVID-19 Follow-Up Study v4.01

34. Please indicate which symptoms remain ongoing. Mark all that apply.

- [ ] Congestion/runny nose
- [ ] Cough
- [ ] Shortness of breath
- [ ] Palpitations (heart racing or pounding)
- [ ] Sore throat
- [ ] Chest pain/pressure
- [ ] NONE OF THE ABOVE

35. Please indicate how long these symptoms lasted or have been ongoing.

| | Less than 2 months | 2 to 3 months | 4 to 5 months | 6 to 8 months | 9 to 12 months | More than 12 months |
|---|---|---|---|---|---|---|
| Congestion/runny nose | ○ | ○ | ○ | ○ | ○ | ○ |
| Cough | ○ | ○ | ○ | ○ | ○ | ○ |
| Shortness of breath | ○ | ○ | ○ | ○ | ○ | ○ |
| Palpitations (heart racing or pounding) | ○ | ○ | ○ | ○ | ○ | ○ |
| Sore throat | ○ | ○ | ○ | ○ | ○ | ○ |
| Chest pain/pressure | ○ | ○ | ○ | ○ | ○ | ○ |

American Red Cross

## American Red Cross COVID-19 Follow-Up Study v4.01

36. Since the start of the pandemic in March 2020, have you had any of these symptoms lasting <u>over 4 consecutive weeks</u> that you did not usually have before? Mark all that apply.

- [ ] Bruising/bleeding easily
- [ ] Change in menstruation cycle
- [ ] Fever or chills
- [ ] Hair loss
- [ ] Joint swelling
- [ ] Joint/muscle pain
- [ ] Skin changes
- [ ] Unintentional weight loss
- [ ] Change in taste
- [ ] Change in smell
- [ ] NONE OF THESE

37. Please indicate which symptoms remain ongoing. Mark all that apply.

- ☐ Bruising/bleeding easily
- ☐ Change in menstruation cycle
- ☐ Fever or chills
- ☐ Hair loss
- ☐ Joint swelling
- ☐ Joint/muscle pain
- ☐ Skin changes
- ☐ Unintentional weight loss
- ☐ Change in taste
- ☐ Change in smell
- ☐ NONE OF THE ABOVE

38. Please indicate how long these symptoms lasted or have been ongoing.

| | Less than 2 months | 2 to 3 months | 4 to 5 months | 6 to 8 months | 9 to 12 months | More than 12 months |
|---|---|---|---|---|---|---|
| Bruising/bleeding easily | ○ | ○ | ○ | ○ | ○ | ○ |
| Change in menstruation cycle | ○ | ○ | ○ | ○ | ○ | ○ |
| Fever or chills | ○ | ○ | ○ | ○ | ○ | ○ |
| Hair loss | ○ | ○ | ○ | ○ | ○ | ○ |
| Joint swelling | ○ | ○ | ○ | ○ | ○ | ○ |
| Joint/muscle pain | ○ | ○ | ○ | ○ | ○ | ○ |
| Skin changes | ○ | ○ | ○ | ○ | ○ | ○ |
| Unintentional weight loss | ○ | ○ | ○ | ○ | ○ | ○ |
| Change in taste | ○ | ○ | ○ | ○ | ○ | ○ |
| Change in smell | ○ | ○ | ○ | ○ | ○ | ○ |

American Red Cross

## American Red Cross COVID-19 Follow-Up Study v4.01

39. Since the start of the pandemic in March 2020, have you had any of these symptoms lasting over 4 consecutive weeks that you did not usually have before? Mark all that apply.

- ☐ Anxiety
- ☐ Depression
- ☐ Post-traumatic stress disorder (PTSD)
- ☐ Change in mood
- ☐ NONE OF THESE

American Red Cross

## American Red Cross COVID-19 Follow-Up Study v4.01

40. Please indicate which symptoms remain ongoing. Mark all that apply.

- ☐ Anxiety
- ☐ Depression
- ☐ Post-traumatic stress disorder (PTSD)
- ☐ Change in mood
- ☐ NONE OF THE ABOVE

41. Please indicate how long these symptoms lasted or have been ongoing.

| | Less than 2 months | 2 to 3 months | 4 to 5 months | 6 to 8 months | 9 to 12 months | More than 12 months |
|---|---|---|---|---|---|---|
| Anxiety | ○ | ○ | ○ | ○ | ○ | ○ |
| Depression | ○ | ○ | ○ | ○ | ○ | ○ |
| Post-traumatic stress disorder (PTSD) | ○ | ○ | ○ | ○ | ○ | ○ |
| Change in mood | ○ | ○ | ○ | ○ | ○ | ○ |

American Red Cross

## American Red Cross COVID-19 Follow-Up Study v4.01

42. For any of the symptoms lasting more than 4 consecutive weeks, did you ever see a doctor, nurse, or other health professional (including in-person and virtual visits)?

- ◯ Yes
- ◯ No
- ◯ I don't know/am unsure

American Red Cross

## American Red Cross COVID-19 Follow-Up Study v4.01

43. What kind(s) of doctor(s), nurse(s), or other health professional(s) did you see for the symptoms lasting more than 4 consecutive weeks? Mark all that apply.

- ☐ Primary care/general/family practitioner
- ☐ Cardiologist
- ☐ Dermatologist
- ☐ Mental health practitioner (counselor, psychologist, psychiatrist, etc)
- ☐ Neurologist
- ☐ Physical/occupational therapist
- ☐ Pulmonologist
- ☐ I don't know/am unsure
- ☐ Other health professional (please specify)

____________________

44. Do you attribute any of the symptom(s) lasting over 4 consecutive weeks to COVID-19 infection?

- ◯ Yes
- ◯ No
- ◯ I don't know/am unsure

45. After your first COVID-19 infection, how long did it take to return to your usual health?

- Less than 1 month
- 1 to 2 months
- 3 to 5 months
- 6 to 8 months
- 9 to 12 months
- More than 12 months
- Have not returned to usual health
- I don't know/am unsure

American Red Cross

## American Red Cross COVID-19 Follow-Up Study v4.01

46. In the past 4 weeks, would you say that in general your health was:

- Excellent
- Very good
- Good
- Fair
- Poor

47. In the past 4 weeks, did poor physical or mental health keep you from doing your usual activities, such as self-care, work, or recreation?

- Yes, often
- Yes, sometimes
- No
- I don't know/am unsure

48. How does your current physical health compare with your physical health prior to the pandemic?

- Better than before the pandemic
- Worse than before the pandemic
- About the same
- I don't know/am unsure

49. How does your current mental health compare with your mental health prior to the pandemic?

- Better than before the pandemic
- Worse than before the pandemic
- About the same
- I don't know/am unsure

American Red Cross

## American Red Cross COVID-19 Follow-Up Study v4.01

50. Which of the following activities would you have been comfortable engaging in over the past month (from today) if permitted? Select all that apply.

- Domestic air travel
- International air travel
- Gathering indoors with friends or family who do not live with me
- Dining indoors at restaurants
- Going to indoor bars or nightclubs
- Going to indoor public places without a mask or face covering
- Attending in-person indoor religious services
- Attending outdoor events or concerts
- Attending indoor events, concerts, or shows
- NONE OF THESE

51. Looking forward, what activities do you anticipate being comfortable engaging in next month if permitted? Select all that apply.

- Domestic air travel
- International air travel
- Gathering indoors with friends or family who do not live with me
- Dining indoors at restaurants
- Going to indoor bars or nightclubs
- Going to indoor public places without a mask or face covering
- Attending in-person indoor religious services
- Attending outdoor events or concerts
- Attending indoor events, concerts, or shows
- NONE OF THESE

52. In the next month, if you anticipate engaging in new or more activities (such as travel, public events, gatherings with friends, indoor dining) compared to last month, what are the reasons? Select all that apply.

- ☐ I expect to be vaccinated or have been recently vaccinated
- ☐ I expect enough other people will be vaccinated
- ☐ I expect the number of new cases to decrease
- ☐ I expect facilities / restaurants / gyms to implement proper guidelines
- ☐ I expect policy changes at the local / state / national level
- ☐ I am willing to tolerate the risks for my personal well-being and mental health
- ☐ I don't expect to engage in new or more activities in the next month

53. In the next month, if you anticipate engaging in fewer activities (such as travel, public events, gatherings with friends, indoor dining) compared to last month, what are the reasons? Select all that apply.

- ☐ I am concerned about decreased immune protection against COVID-19
- ☐ I am concerned about SARS-CoV-2 variants
- ☐ I don't think enough other people are vaccinated
- ☐ I am concerned about current or future increase in cases
- ☐ I don't think facilities / restaurants / gyms are implementing proper restrictions
- ☐ I am concerned about low levels of mask use in public places
- ☐ I don't expect to engage in fewer activities next month

**Thank you for clicking through to learn more about our American Red Cross (ARC) survey!**

**You are eligible for this survey because you donated blood between June 15, 2020 and December 31, 2021, had one or more of these donations tested for antibodies to the virus that causes COVID-19 (SARS-CoV-2), and you completed a previous survey on your experience with COVID-19.**

**We appreciate your support of Red Cross research and are now inviting you to participate in an abbreviated follow-up survey on COVID-19. The purpose of the current survey is to better understand the association between donor antibodies to the COVID-19 virus and risk for subsequent infection and illness from SARS-CoV-2. Although you have previously completed one of our surveys on COVID-19, your participation in this survey is still valuable to help answer current scientific questions.**

**Please note that in this survey, we are not asking about your cumulative history with COVID-19 infection and vaccinations. Rather, we are asking about your experience since you last completed our survey, the date of which will be provided throughout the survey.**

**This research is being conducted in collaboration with the Centers for Disease Control and Prevention. Your participation is voluntary, and there will be no impact to your relationship with American Red Cross if you decide not to participate. Please note that information that can be linked to you will not be shared outside of ARC. If you agree to participate, please begin the survey below.**

**If you have questions or concerns, please contact us Monday through Friday, 8am to 4pm Central time at 855-563-7024.**

1. Since you last completed our survey on {{ contact.custom2 }}, have you been infected by SARS-CoV-2, the virus that causes COVID-19?

(*If you have been infected more than once, please answer for your earliest infection*).

- ○ Yes, and my infection was confirmed by a diagnostic test (nose swab, throat swab, saliva test) or a healthcare provider (or both)
- ○ Yes, but my infection was confirmed only by a blood test (antibody test, such as from Red Cross)
- ○ Yes, I think I have been infected but it has not been confirmed by a test or diagnosis
- ○ No
- ○ I don't know/am unsure

2. Do you think you have been infected by the COVID-19 virus more than once between {{ contact.custom2 }} and today?

- ○ Yes, twice
- ○ No
- ○ I don't know/am unsure

3. When were you first infected by the COVID-19 virus between {{ contact.custom2 }} and today?
Please approximate if unsure.

| | Month | Day | Year |
|---|---|---|---|
| Date of first COVID-19 infection between {{ contact.custom2 }} and today | | | |

4. When you were first infected by the COVID-19 virus between {{ contact.custom2 }} and today, did you have any symptoms?

- ○ Yes
- ○ No
- ○ I don't know/am unsure

## American Red Cross COVID-19 Follow-Up Study_Short.FQ2

5. In the 2 weeks after your COVID-19 symptoms started, which of the following symptoms did you have? (This refers to the first time you were infected with the COVID-19 virus between {{ contact.custom2 }} and today). Mark all that apply.

- ☐ Fever
- ☐ Chills or rigors (shaking chills)
- ☐ Muscle aches
- ☐ Headache
- ☐ Sore throat
- ☐ Nausea or vomiting
- ☐ Diarrhea
- ☐ Fatigue
- ☐ Congestion or runny nose
- ☐ Cough
- ☐ Shortness of breath or difficulty breathing
- ☐ Loss of smell
- ☐ Loss of taste
- ☐ Confusion
- ☐ Persistent chest pain or chest pressure
- ☐ Pale, gray, or blue-colored skin, lips or nail beds
- ☐ Difficulty waking up or staying awake
- ☐ Other (please specify)

6. Were you hospitalized for COVID-19 for your first infection between {{ contact.custom2 }} and today? Choose the answer that best applies.

- ◯ Yes, I was hospitalized and admitted to the intensive care unit
- ◯ Yes, I was hospitalized but not admitted to the intensive care unit
- ◯ No, I was not hospitalized
- ◯ I don't know/am unsure

## American Red Cross COVID-19 Follow-Up Study_Short.FQ2

7. When do you think you were infected by the COVID-19 virus for the second time between {{ contact.custom2 }} and today?
Please approximate if unsure.

| | Month | Day | Year |
|---|---|---|---|
| Date of second COVID-19 infection between {{ contact.custom2 }} and today | | | |

8. Was your second infection from the COVID-19 virus between {{ contact.custom2 }} and today confirmed?

- ◯ Yes, and my infection was confirmed by a diagnostic test (nose swab, throat swab, saliva test) or a healthcare provider (or both)
- ◯ No. I think I have been infected but it has not been confirmed by a test or diagnosis.
- ◯ I don't know/am unsure

9. When you had your second infection with the COVID-19 virus between {{ contact.custom2 }} and today, did you have any symptoms?

- ◯ Yes
- ◯ No
- ◯ I don't know/am unsure

## American Red Cross COVID-19 Follow-Up Study_Short.FQ2

10. In the 2 weeks after your COVID-19 symptoms started, which of the following symptoms did you have? (This refers to the second time you were infected with the COVID-19 virus between {{ contact.custom2 }} and today). Mark all that apply.

- [ ] Fever
- [ ] Chills or rigors (shaking chills)
- [ ] Muscle aches
- [ ] Headache
- [ ] Sore throat
- [ ] Nausea or vomiting
- [ ] Diarrhea
- [ ] Fatigue
- [ ] Congestion or runny nose
- [ ] Cough
- [ ] Shortness of breath or difficulty breathing
- [ ] Loss of smell
- [ ] Loss of taste
- [ ] Confusion
- [ ] Persistent chest pain or chest pressure
- [ ] Pale, gray, or blue-colored skin, lips or nail beds
- [ ] Difficulty waking up or staying awake
- [ ] Other (please specify)

11. Were you hospitalized for your second COVID-19 infection between {{ contact.custom2 }} and today? Choose the answer that best applies.

- ○ Yes, I was hospitalized and admitted to the intensive care unit
- ○ Yes, I was hospitalized but not admitted to the intensive care unit
- ○ No, I was not hospitalized
- ○ I don't know/am unsure

## American Red Cross COVID-19 Follow-Up Study_Short.FQ2

12. Have you received any COVID-19 vaccines, including boosters between {{ contact.custom2 }} and today?
(*Please note you have previously provided information on vaccine doses, including boosters, that occurred prior to {{ contact.custom2 }} and we will connect the updated information with that you previously gave.)*

- ○ Yes, one dose
- ○ Yes, two doses
- ○ No, but I plan to
- ○ No, and I don't plan to
- ○ I don't know/am unsure

## American Red Cross COVID-19 Follow-Up Study_Short.FQ2

13. For your first vaccine dose between {{ contact.custom2 }} and today, which COVID-19 vaccine did you receive?

- ◯ Pfizer-BioNTech
- ◯ Johnson and Johnson
- ◯ Moderna
- ◯ I do not know which vaccine I received
- ◯ Other (please specify)

14. What was the date of your first vaccine dose between {{ contact.custom2 }} and today? Please approximate if unsure.

| | Month | Day | Year |
|---|---|---|---|
| Date of first vaccine dose between {{ contact.custom2 }} and today | | | |

15. For your second vaccine dose between {{ contact.custom2 }} and today, which COVID-19 vaccine did you receive?

- ◯ Pfizer-BioNTech
- ◯ Johnson and Johnson
- ◯ Moderna
- ◯ I do not know which vaccine I received
- ◯ Other (please specify)

16. What was the date of your second vaccine dose between {{ contact.custom2 }} and today? Please approximate if unsure.

| | Month | Day | Year |
|---|---|---|---|
| Date of second vaccine dose between {{ contact.custom2 }} and today | | | |

# Understanding COVID-19 Baseline Survey

**We are going to ask you two initial questions. Depending on your answers to these questions we may ask you additional questions to collect more information from you. We will also ask you about your general health today in the last part of the survey.**

**COVID-19 Infection**

1. Outside of COVID-19 antibody testing of your blood donation(s), have you ever had a positive COVID-19 result based on a swab test (a sample was collected from inside your nose or throat), or a saliva (spit) sample, or have you been diagnosed by a physician as having COVID-19?
    - o Yes
    - o No

**SARS-CoV-2 Vaccination**

2. Have you received a COVID-19 vaccine? This is also known as a SARS-CoV-2 vaccine.
    - o Yes
    - o No

If 1. = Yes, go to 3.

If 2. = Yes, go to 13.

If 1 = No and 2 = No, go to 19.

**COVID-19 Testing and Symptom History**

**Since you indicated you have had a positive test for COVID-19, we are going to ask you more questions about COVID-19 testing and symptoms you may have had.**

3. When did you first have a positive COVID-19 test? [Select Date: Month_Day_Year]

4. Which type of COVID-19 test did you have?
    - Virus Test. If you have a laboratory report it may call this test a Nucleic Acid Amplification Test (NAAT) or a Polymerase Chain Reaction (PCR) Test.

    - Antigen Test

        If selected: Do you know if this was a rapid test where the results were available to you within 20 minutes?

        - Yes
        - No
        - Unsure

    - Antibody Test. Please don't include testing done from your blood donation(s) when answering.

        If selected: Do you know if this was a rapid test where the results were available to you within 20 minutes?

        - Yes
        - No
        - Unsure
    - Unsure

5. Have you had more than one positive COVID-19 test?
    - Yes Go to 6.
    - No Go to Q8
    - Unsure Go to Q8

6. When did you have your second positive COVID-19 test? [Select Date: Month_Day_Year]

7. Which type of COVID-19 test did you have?
    - Nucleic Acid Amplification Test (NAAT). This is also known as a Polymerase Chain Reaction (PCR) Test

    - Antigen Test

        If selected: Do you know if this was a rapid test where the results were available to you within 20 minutes?

        - Yes

- No
- Unsure

- Antibody

  If selected: Do you know if this was a rapid test where the results were available to you within 20 minutes?

  - Yes
  - No
  - Unsure

- Unsure

8. Did you have any symptoms before or after (either of) your positive COVID-19 test(s)?

   - Yes
   - No
   - Unsure

9. a. Select the symptoms that you had around the time of your FIRST (or only) positive COVID-19 test. Please do not include possible reactions following vaccination when answering these questions.

   b. Select the symptoms that you had around the time of your SECOND positive COVID-19 test. Please do not include possible reactions following vaccination when answering these questions.

| | Experienced? | | | If Yes, how many episodes did you have? A symptom episode lasts several days in a row. | How many days did the average episode last? |
|---|---|---|---|---|---|
| | Yes | No | Unsure | | |
| Cough | | | | | |
| Shortness of breath or difficulty breathing | | | | | |
| Sore throat | | | | | |
| Runny nose | | | | | |
| Loss of taste or smell | | | | | |
| Fever | | | | | |
| Headache | | | | | |
| Muscle or body aches | | | | | |
| Nausea, vomiting, or diarrhea | | | | | |
| Extreme fatigue | | | | | |
| Trouble concentrating, also known as “brain fog” | | | | | |
| Problems with memory | | | | | |

10. Did you seek healthcare at any of these because of your illness?
    a. Hospital/ER
    b. Urgent care
    c. Physician’s office or clinic
    d. Telehealth
    e. Other
    f. Did not seek healthcare

11. Were you hospitalized because of your illness?
    a. Yes
    b. No

12. a. Would you describe yourself as having had ‘long COVID’, that is, did you experience symptoms that lasted for at least four weeks after being first infected that were not explained by something else?
    - Yes
    - No
    - Unsure

    If yes:
    b. Are these ‘long COVID’ symptoms still occurring today?
    - Yes
    - No
    - Unsure

**SARS-CoV-2 Vaccination Details**

**You indicated you have been vaccinated for COVID-19. To answer the next questions, it will be very helpful if you have your vaccination card in front of you. This is the card that was given to you by the person who gave you the vaccine.**

13. Please refer to your vaccination card to provide the following:

| | Received | | If received, please select Vaccine Name | If Other: Enter name of other vaccine | Date Received: Month | Date Received: Day | Date Received: Year |
|---|---|---|---|---|---|---|---|
| | Yes | No | | | | | |
| COVID-19 Vaccine First Dose | | | | | | | |
| COVID-19 Vaccine Second Dose | | | | | | | |
| Dose of the Same Vaccine or Booster | | | | | | | |

14. What motivated you to get vaccinated? Select all that apply.

- ☐ Protect my health
- ☐ Protect health of family/friends
- ☐ Protect health of co-workers/community
- ☐ To get back to work/school
- ☐ To resume social activities
- ☐ To resume travel
- ☐ Because others encouraged me to get vaccinated
- ☐ Not sure
- ☐ Other

15. Did you have a reaction following any of your COVID-19 vaccinations?
    - ○ Yes
    - ○ No
    - ○ Unsure

16. Select the reaction you had after your FIRST COVID-19 vaccination:
Select the reaction you had after your SECOND COVID-19 vaccination:
Select the reaction you had after your OTHER COVID-19 vaccination or BOOSTER:

| | Experienced? | | | If Yes, for how long? | | |
|---|---|---|---|---|---|---|
| | Yes | No | Unsure | Less than 1 day | 1 to 3 days | 4 days or more |
| Soreness, redness, or swelling at injection site | | | | | | |
| Fever or chills | | | | | | |
| Muscle pain or any other body aches | | | | | | |
| Fatigue | | | | | | |
| Nausea | | | | | | |

**Opinions on Vaccination**

**The next questions cover your opinions about vaccination. There are no right answers, please tell us what you think:**

**If 2 = Yes, ask 17, 18, and 22:**

17. How concerned were you about getting a COVID-19 vaccine?
    - Not at all concerned
    - A little concerned
    - Moderately concerned
    - Very concerned
    - Not sure

18. How easy was it to get a COVID-19 vaccine for yourself?
    - Very easy
    - Somewhat easy
    - Somewhat difficult
    - Very difficult
    - Not sure

**If 2 = No, ask 19-22:**

19. How concerned are you about getting a COVID-19 vaccine?

    - Not at all concerned
    - A little concerned
    - Moderately concerned
    - Very concerned
    - Not sure

20. What makes you not want to get a COVID-19 vaccine? Select all that apply.
    - ◻ Unsure the vaccines are safe.
    - ◻ Personal, cultural, or religious beliefs against vaccination.
    - ◻ I am young and do not need to worry about being vaccinated.
    - ◻ I am worried about being exposed to SARS-CoV-2 when I am at the vaccination location.
    - ◻ I can't go on my own (I have a physical or other limitation) or don't have transportation.
    - ◻ I don't know where to go to get vaccinated.
    - ◻ I'm not eligible to get a COVID-19 vaccine or have a medical reason that makes me ineligible to get vaccinated (e.g., I have had a severe allergy to vaccines in the past).
    - ◻ The vaccination clinic is too far away or the hours of operation are inconvenient.
    - ◻ The waiting time is too long. It is difficult to find or make an appointment.
    - ◻ I am too busy to get vaccinated.
    - ◻ It is difficult to arrange for childcare.
    - ◻ I don't have time off work.
    - ◻ Other
    - ◻ Not sure

21. How easy would it be to get a COVID-19 vaccine for yourself?

    - Very easy
    - Somewhat easy
    - Somewhat difficult
    - Very difficult
    - Not sure

22. How likely are you to recommend getting the COVID-19 vaccine to others?

    - Not at all likely
    - Somewhat likely
    - Extremely likely
    - Not sure

**Medical History Information**

**The final section of the survey will ask you about other parts of your medical history. These data will help us to understand whether other health-related topics influence the chance of getting SARS-CoV-2 or COVID-19.**

23. On the scale below showing 0 to 10, please rate your current health today by dragging or touching the line below. 0 is the worst possible health you can imagine and 10 is the best possible health you can imagine.

●————————————————●

0 1 2 3 4 5 6 7 8 9 10

24. Have you had, or do you currently have any of the following health conditions? Check all that apply.
    - ◻ Asthma
    - ◻ Other respiratory disease
    - ◻ Heart disease or other cardiovascular disease
    - ◻ High blood pressure
    - ◻ Diabetes
    - ◻ Any kind of immune system disorder
    - ◻ Kidney disease
    - ◻ Liver disease
    - ◻ Neurological disease
    - ◻ Cancer
    - ◻ None of the above

25. During the past 30 days, have you smoked part or all of a cigarette?
    - ○ Yes
    - ○ No

**If 25=Yes, ask 25a.**

25a. On average, during the past 30 days, how many cigarettes did you smoke per day?
- ○ Zero
- ○ More than zero, but less than one cigarette
- ○ 1 cigarette
- ○ 2 to 5 cigarettes
- ○ 6 to 15 cigarettes
- ○ 16 to 25 cigarettes
- ○ 26 to 35 cigarettes
- ○ More than 35 cigarettes
- ○ Not sure

**Submission Confirmation Page**

**If consented:**
You have reached the end of the survey.

Thank you for taking the time to provide these answers to us. We truly appreciate your donations and willingness to be a part of research conducted at Vitalant Research Institute. We will send you a survey about every three months to update your information as part of your participation in this research. You may opt out at any time by declining the next survey invitation.

**If did not consent:**
You have reached the end of the survey.

**END**

# Understanding COVID-19 Follow-up Survey

**We are going to ask you two initial questions. The answers to these may have changed since your last survey on [DATE]. Depending on your answers to these questions we may ask you additional questions to collect more information from you. We will also ask you about your general health today in the last part of the survey.**

**COVID-19 Infection**

1. Outside of COVID-19 antibody testing of your blood donation(s), **since your last survey** have you had a positive COVID-19 result based on a swab test (a sample was collected from your inside your nose or throat), a saliva (spit) sample, or been diagnosed by a physician as having COVID-19?

   - o Yes
   - o No

**SARS-CoV-2 Vaccination**

2. **Since your last survey** have you received any new or additional doses of a COVID-19 vaccine? This is also known as a SARS-CoV-2 vaccine.

   - o Yes
   - o No

If 1. = Yes, go to 3.

If 2. = Yes, go to 14.

If 1 = No and 2 = No, go to 18.

**COVID-19 Testing and Symptom History**

**Since you indicated you have had a positive test or physician diagnosis for COVID-19 since your last survey on [DATE], we are going to ask you more questions about COVID-19 testing and symptoms you may have had.**

3. How many positive COVID-19 tests or physician diagnoses have you had since your last survey?
   - o 1
   - o 2
   - o 3
   - o 4
   - o 5 or more

The questions below are in regard to your FIRST (or only) positive COVID-19 test or physician diagnosis since your last survey on [DATE].

If more than one in Q3:
The questions below are in regard to your [Nth] positive COVID-19 test or physician diagnosis since your last survey.

4a. When did you have your first positive COVID-19 test or physician diagnosis since your last survey?
[Select Date: Month_Day_Year]

If more than one in Q3:
4b-n. When did you have your [nth] positive COVID-19 test or physician diagnosis?

5a. Which type of COVID-19 test did you have?

If more than one:
5b-n. Which type of COVID-19 test did you have?

- o Virus Test. If you have a laboratory report it may call this test a Nucleic Acid Amplification Test (NAAT) or a Polymerase Chain Reaction (PCR) Test.
- o Antigen Test

  If selected: Do you know if this was a rapid test where the results were available to you within 20 minutes?

  - o Yes
  - o No
  - o Unsure

- Antibody Test. Please don't include testing done from your blood donation(s) when answering.

  If selected: Do you know if this was a rapid test where the results were available to you within 20 minutes?

  - Yes
  - No
  - Unsure

- Diagnosed by physician

- Unsure

6a. Did you have any symptoms around the time of your positive COVID-19 test or physician diagnosis?

If more than one in Q3:
6b-n. Did you have any symptoms around the time of your positive COVID-19 test or physician diagnosis?

- Yes
- No
- Unsure

  Yes: Go to Q7

  No: Go to 15

7a. Select the symptoms that you had around the time of your positive COVID-19 test or physician diagnosis. Please do not include possible reactions following vaccination when answering these questions.

If more than one in Q3:
7b-n. Select the symptoms that you had around the time of your positive COVID-19 test or physician diagnosis. Please do not include possible reactions following vaccination when answering these questions.

| | Experienced? | | | If Yes, how many episodes did you have? A symptom episode lasts several days in a row. | How many days did the average episode last? |
|---|---|---|---|---|---|
| | Yes | No | Unsure | | |
| Cough | | | | | |
| Shortness of breath or difficulty breathing | | | | | |
| Sore throat | | | | | |
| Congestion or runny nose | | | | | |
| Loss of taste or smell | | | | | |
| Fever | | | | | |
| Headache | | | | | |
| Muscle or body aches | | | | | |
| Nausea, vomiting, or diarrhea | | | | | |
| Extreme fatigue | | | | | |
| Trouble concentrating, also known as “brain fog” | | | | | |
| Problems with memory | | | | | |
| Other: [In-line open text box] | | | | | |

8a. Did you seek healthcare at any of these because of this illness?

If more than one in Q3:
8b-n. Did you seek healthcare at any of these because of this illness?

- ◻ Hospital/ER
- ◻ Urgent care
- ◻ Physician’s office or clinic
- ◻ Telehealth
- ◻ Other: [In-line open text box]
- ◻ Did not seek healthcare

9a. Were you hospitalized because of this illness?

If more than one in Q3:
9b-n. Were you hospitalized because of this illness?

- ○ Yes
- ○ No

The next questions ask about potential long COVID symptoms. Also known as post-acute sequelae of SARS-CoV-2 infection (PASC), long COVID refers to a range of symptoms that can last for months after first being infected with SARS-CoV-2. They can even first appear weeks after the infection has resolved. Long COVID can happen to anyone infected with SARS-CoV-2, even if the illness was mild or entirely asymptomatic.

10. Did you experience symptoms that lasted for at least four weeks any time after being first infected with SARS-CoV-2 that were not explained by something else?
    - o Yes
    - o No

11. Please mark any symptoms lasting 4 or more weeks that you may have had, indicating how long these symptoms lasted and if they are still ongoing.

| | Experienced? | | | If Yes, for how long did symptoms last? [<2 months; 2-3 months; 4-5 months; 6-8 months; 9-12 months; More than 12 months] | If Yes, is this symptom still ongoing? | | |
|---|---|---|---|---|---|---|---|
| | Yes | No | Unsure | | Yes | No | Unsure |
| Fatigue/weakness | | | | | | | |
| Headache | | | | | | | |
| Symptoms that get worse after physical or mental activities (post-exertional malaise) | | | | | | | |
| Problems sleeping | | | | | | | |
| Problems speaking or communicating | | | | | | | |
| Problems with balance/movement | | | | | | | |
| Tingling/numbness in any part of body | | | | | | | |
| Difficulty thinking or concentrating (sometimes referred to as “brain fog”) | | | | | | | |
| Dizziness/light headedness/fainting | | | | | | | |
| Problems swallowing or chewing | | | | | | | |

| | | | | | |
|---|---|---|---|---|---|
| Appetite changes | | | | | |
| Constipation | | | | | |
| Diarrhea | | | | | |
| Nausea/vomiting | | | | | |
| Stomach pain | | | | | |
| Congestion/runny nose | | | | | |
| Cough | | | | | |
| Shortness of breath | | | | | |
| Palpitations (heart racing or pounding) | | | | | |
| Sore throat | | | | | |
| Chest pain/pressure | | | | | |
| Bruising/bleeding easily | | | | | |
| Change in menstruation cycle | | | | | |
| Fever or chills | | | | | |
| Hair loss | | | | | |
| Joint swelling | | | | | |
| Joint/muscle pain | | | | | |
| Skin changes | | | | | |
| Unintentional weight loss | | | | | |
| Change in taste | | | | | |
| Change in smell | | | | | |
| Anxiety | | | | | |
| Depression | | | | | |
| Post-traumatic stress disorder (PTSD) | | | | | |
| Change in mood | | | | | |
| Other: [In-line open text box] | | | | | |

12. a. For any of the symptoms lasting more than 4 consecutive weeks, did you ever see a doctor, nurse, or other health professional (including in-person and virtual visits)?
    - o Yes
    - o No
    - o I don’t know/unsure

    b. If yes: What kind(s) of doctor(s), nurse(s) or other health professional(s) did you see for the symptoms(s) lasting more than 4 consecutive weeks (check all that apply)?

    - o Primary care/general/family practitioner
    - o Cardiologist
    - o Dermatologist

- Mental health practitioner (counselor, psychologist, psychiatrist, etc.)
- Neurologist
- Physical/occupational therapist
- Pulmonologist
- I don’t know/am unsure
- Other health professional (please specify): [In-line open text box]

13. a. Would you describe yourself as having ‘long COVID’, that is, do you attribute any of the symptom(s) lasting more than 4 weeks to COVID-19 infection?
    - Yes
    - No
    - Unsure

    b. If yes: Are these ‘long COVID’ symptoms still occurring today?
    - Yes
    - No
    - Unsure

**SARS-CoV-2 Vaccination Details**

**You indicated you have been vaccinated for COVID-19 since your last survey on [DATE]. To answer the next questions, it will be very helpful if you have your vaccination card in front of you. This is the card that was given to you by the person who gave you the vaccine.**

14. Please refer to your vaccination card to provide the following for any vaccines or boosters received after you completed your last survey.

| | What vaccine dose was this? Drop-down: <br>• First COVID-19 Vaccine Dose <br>• Second COVID-19 Vaccine Dose <br>• Third COVID-19 Vaccine Dose or Booster <br>• Fourth COVID-19 Vaccine Dose or Booster <br>• Nth COVID-19 Vaccine Dose or Booster | Select Vaccine Name Drop-down: <br>• Pfizer-BioNTech <br>• Moderna <br>• Janssen, Johnson & Johnson, or J&J <br>• Novavax <br>• AstraZeneca-Oxford <br>• Other | If Other: | Date Received : Month | Date Received : Day | Date Received : Year |
|---|---|---|---|---|---|---|
| Additional COVID-19 Vaccination 1 | | | | | | |
| Additional COVID-19 Vaccination 2 | | | | | | |
| Additional COVID-19 Vaccination 3 | | | | | | |
| … | | | | | | |
| Additional COVID Vaccination N | | | | | | |

15. a. What motivated you to get vaccinated? Select all that apply.

- ◻ Protect my health
- ◻ Protect health of family/friends

- ☐ Protect health of co-workers/community
- ☐ To get back to work/school
- ☐ To resume social activities
- ☐ To resume travel
- ☐ Because others encouraged me to get vaccinated
- ☐ Not sure
- ☐ Other: [In-line open text box]

16. Did you have a reaction following any of your COVID-19 vaccinations?
    - o Yes
    - o No
    - o Unsure

17. a. Select the reaction you had after your FIRST additional COVID-19 vaccination:
    b. Select the reaction you had after your SECOND additional COVID-19 vaccination:
    c. Select the reaction you had after your THIRD additional COVID-19 vaccination:
    …
    n. Select the reaction you had after your Nth additional COVID-19 vaccination:

| | Experienced? | | | If Yes, for how long? | | |
|---|---|---|---|---|---|---|
| | Yes | No | Unsure | Less than 1 day | 1 to 3 days | 4 days or more |
| Soreness, redness, or swelling at injection site | | | | | | |
| Fever or chills | | | | | | |
| Muscle pain or any other body aches | | | | | | |
| Fatigue | | | | | | |
| Nausea | | | | | | |
| Swollen lymph nodes in armpit or near collar bone on the side of body where vaccine was administered | | | | | | |
| Other: [In-line open text box] | | | | | | |

**Medical History Information**

**The final question of the survey asks about your general health today. These data will help us to understand whether other health-related topics influence the chance of getting SARS-CoV-2 or COVID-19.**

18. On the scale below showing 0 to 10, please rate your current health today by dragging or touching the line below. 0 is the worst possible health you can imagine and 10 is the best possible health you can imagine.

0 1 2 3 4 5 6 7 8 9 10

**If consented:**
You have reached the end of the survey.

Thank you for taking the time to provide these answers to us. We truly appreciate your donations and willingness to be a part of research conducted at Vitalant Research Institute. We will send you a survey about every three months to update your information as part of your participation in this research. You may opt out at any time by declining the next survey invitation.

**If did not consent:**
You have reached the end of the survey.

**END**
